# Deep sequencing of 16 *Ixodes ricinus* ticks unveils insights into their interactions with endosymbionts

**DOI:** 10.1128/msystems.00507-25

**Published:** 2025-06-16

**Authors:** Paulina M. Lesiczka, Tal Azagi, Aleksandra I. Krawczyk, William T. Scott, Ron P. Dirks, Ladislav Simo, Gerhard Dobler, Bart Nijsse, Peter J. Schaap, Hein Sprong, Jasper J. Koehorst

**Affiliations:** 1 Department of Veterinary Sciences, Faculty of Agrobiology, Food and Natural Resources, Czech University of Life Sciences 234829 , Prague, Prague, Czechia; 2 NVWA-Centrum Monitoring Vectoren, Wageningen, the Netherlands; 3 Centre for Infectious Diseases (CIb), National Institute for Public Health and the Environment (RIVM) 10206, Bilthoven, Utrecht, the Netherlands; 4 Laboratory of Systems and Synthetic Biology, Wageningen University & Research 685494, Wageningen, Gelderland, the Netherlands; 5 UNLOCK, Wageningen University & Research 4508, Wageningen, Gelderland, the Netherlands; 6 Future Genomics Technologies BV, Leiden, the Netherlands; 7 Laboratoire de Santé Animale, École Nationale Vétérinaire d’Alfort, UMR BIPAR, Anses INRAE 355169 , Maisons-Alfort, France; 8 Bundeswehr Institute of Microbiology 539152, Munich, Bavaria, Germany; University of Connecticut, Storrs, Connecticut, USA

**Keywords:** *Ixodes ricinus*, deep sequencing, symbionts, genome reconstruction, *Midichloria mitochondrii*, *Rickettsia helvetica*, genome-scale metabolic modeling, cophylogenetic analysis, paternal transmission

## Abstract

*Ixodes ricinus* ticks act as vectors for numerous pathogens that present substantial health threats. Additionally, they harbor vertically transmitted symbionts, some of which have been linked to diseases. The difficulty of isolating and cultivating these symbionts has hampered our understanding of their biological role, their potential to cause disease, and their modes of transmission. To expand our understanding of the tick symbiont *Midichloria mitochondrii* and *Rickettsia helvetica*, which has been linked to disease in humans, we utilized deep sequencing on 16 individual adult female ticks collected from coastal dune and forested areas in the Netherlands. By employing a combination of second- and third-generation sequencing techniques, we successfully reconstructed the complete genomes of *M. mitochondrii* from 11 individuals, *R. helvetica* from eight individuals, and the mitochondrial genome from all ticks. Additionally, we visualized the location of *R. helvetica* in tick organs and constructed genome-scale metabolic models (GEMs) of both symbionts to study their environmental dependencies. Our analysis revealed a strong cophylogeny between *M. mitochondrii* and mitochondrial genomes, suggesting frequent maternal transmission. In contrast, the absence of cophylogeny between *R. helvetica* and the mitochondrial genomes, coupled with its presence in the receptaculum seminis of *I. ricinus* females, raises the possibility of paternal transmission of *R. helvetica*. Notably, the genetic diversity of *R. helvetica* was found to be very low, except for the rickA virulence gene, where the presence of up to 13 insertions of a 33 nt-long repeat led to significant variability. However, this variation could not account for the differences in infection prevalence observed across eight distinct locations in the Netherlands. By employing deep sequencing, it becomes feasible to extract complete genomes and genetic data of symbionts directly from their host organisms. This methodology serves as a robust means to gain fresh insights into their interactions. Our observations, which suggest paternal transmission of *R. helvetica*, a relatively unexplored mode of transmission in ticks, require validation through experimental investigations. The genetic variations identified in the rickA virulence gene of *R. helvetica* have the potential to influence the infectivity and transmission dynamics of *R. helvetica*.

**IMPORTANCE:**

Ticks are vectors of numerous human pathogens; however, the microbial interactions within ticks and the mechanisms governing pathogen transmission remain poorly understood. This study uses deep sequencing of individual *Ixodes ricinus* to reconstruct high-quality genomes of endosymbionts and the mitochondrion of the tick, revealing previously undetected microbial dynamics. Notably, we recovered low-abundance *Rickettsia* and *Midichloria* genomes from single ticks and present evidence that suggests paternal transmission of *R. helvetica*. These findings offer novel insights into the ecology and evolution of tick-associated microbes and have implications for understanding the origins and spread of tick-borne diseases.

## INTRODUCTION

*Ixodes ricinus*, the most common tick species in the Palearctic realm, carries a variety of facultative symbionts, some of which can cause infectious diseases in humans. The most common tick-borne diseases associated with *I. ricinus* are Lyme disease (LB) (which is caused by *Borrelia burgdorferi sensu lato*) and tick-borne encephalitis (TBE), which is caused by tick-borne encephalitis virus (1). In addition*, I. ricinus* transmits several emerging pathogens such as *Rickettsia helvetica, Anaplasma phagocytophilum, Borrelia miyamotoi, Neoehrlichia mikurensis*, and several *Babesia* species (2). Infections with these microorganisms have been associated with human diseases in cross-sectional and case studies, but their pathogenic potential has not been fully understood (3, 4). These microorganisms can influence the tick host’s metabolism, development, reproduction, stress defense, and immunity (5) and may indirectly contribute to the transmission dynamics of tick-borne pathogens, significantly impacting human and animal health (6). This implies a broader role within the tick host, and thus, we refer to them as tick symbionts.

Bacterial endosymbionts of ticks are bacteria living within the body or cells of ticks, and their survival is critically dependent on the life cycle of ticks. Some of those bacteria might also have the ability to cause infection in vertebrate hosts, more specifically humans, thereby causing disease. Such bacteria can therefore also be considered (human) pathogens. In other words, “pathogen” and “symbiont” are not mutually exclusive properties/descriptions of bacteria living inside ticks (7).

The biological role, virulence, and transmission dynamics of tick symbionts and some of the pathogens are often difficult to study due to challenges in isolating and culturing them. A notable example is *Midichloria mitochondrii*, initially discovered in the ovaries of ticks. *Midichloria mitochondrii* has been found to be highly prevalent in various tissues of *I. ricinus* (8–11). Since its discovery, *Midichloria* symbionts have been detected in several tick species across different genera, albeit with varying prevalence (12), and are believed to contribute to detoxification, vitamin supply, and energy-related functions, based on their observed interactions and effects within host cells (13). Although *M. mitochondrii* has been detected in the blood of animals, it has yet to be associated with human disease and is mainly considered in the context of tick-symbiont interactions (14), and other symbionts such as *Rickettsia helvetica* cannot be so clearly defined.

*R. helvetica* is a gram-negative, obligate intracellular bacterium formerly included in the spotted fever group rickettsiae (SFGR) (15). *R. helvetica* can be transmitted by tick bites and has been associated with human disease. Only a few human cases have been reported in Europe (16–21), and detection in cerebrospinal fluid of suspected Lyme neuroborreliosis patients has been shown in the Netherlands and Denmark. However, it is unclear whether infection with *R. helvetica* can cause disease or might change the course of Lyme disease (22, 23). Moreover, studies on the involvement of *R. helvetica* in sarcoidosis seem to be contradictory (24). Thus, the extent of *R. helvetica*’s infectivity and pathogenicity is still under investigation, and little is known about its ecoepidemiology (25, 26). DNA of *R. helvetica* has only been sporadically detected in wildlife (27–31) with no clear animal reservoir. This symbiont is transmitted vertically from one generation of ticks to the next and has been repeatedly isolated from questing *I. ricinus* in European countries (32), which are currently thought to be the main reservoir for *R. helvetica* in nature (27). *R. helvetica* infection rates in *I. ricinus* vary from 0% to 66%, resulting in highly variable human exposure from tick bites. The reason for this high variability is unknown and could be attributed to the capacity of *R. helvetica* to be beneficial for the tick host under certain environmental conditions (33), or it could be related to genetic variation within *R. helvetica* populations, but the latter has not been investigated so far.

The pathogenic potential of *R. helvetica* has been explored, to a certain extent, on the cellular level. Although other rickettsial species, which are pathogenic to humans, utilize actin-based cell-to-cell motility (34), a culture of *R. helvetica* has been shown to lack this ability *in vitro* (26), possibly due to a deletion in the *sca*2 gene, encoding a protein required for virulence that stimulates actin filament assembly (35, 36). In addition, although the sequence encoding the RickA protein, a bacterial actin nucleator (37), was complete, its expression was apparently insufficient to enable actin-based motility in the strains studied (26). Comparative analyses of its genetic makeup and metabolic potential are needed to clarify the pathogenic potential of *R. helvetica* as well as its interaction and localization within the tick host.

Advances in sequencing technologies have ushered in a new era of understanding by enabling the comprehensive annotation of complete tick mitochondrial genomes (mt genomes). These genomic insights have significantly enriched our understanding of tick phylogeny, population genetics, and evolutionary processes (38, 39). Given ticks’ intimate association with symbiotic microbes crucial for their hematophagous lifestyle (40), the incorporation of mt genomes into co-phylogeny analyses presents a promising avenue for unraveling the evolutionary patterns of tick symbionts (41). This approach holds particular significance for symbionts like *M. mitochondrii,* which inhabit tick mitochondria offering a unique window into their evolutionary trajectory (42).

Genome-scale model (GEM) reconstruction and analysis can further enhance this understanding by detailing the metabolic interactions and dependencies between ticks and their symbionts. These models can elucidate how these endosymbionts may contribute to the metabolic network, revealing potential targets for controlling tick populations and preventing tick-borne diseases. Additionally, GEMs can provide insights into the co-evolutionary dynamics of ticks and their symbionts, as has been shown in recent work in insects (43), offering a clearer picture of how these complex biological systems have adapted and evolved together over time.

In this study, we applied deep sequencing on 16 *I. ricinus* specimens to unveil the genetic variation and differences in transmission dynamics of *M. mitochondrii* and *R. helvetica*. Complete genomes were obtained directly from the individual ticks using a hybrid assembly approach combining PromethION and Illumina sequencing. In addition to interspecies whole genome comparisons, we explored the genetic association of *R. helvetica* and *M. mitochondrii* with their tick hosts to better understand the mode of transmission of these symbionts. To further our understanding of the latter, we visualized *R. helvetica* in tick organs.

## MATERIALS AND METHODS

### Tick specimen metadata, DNA isolation and sequencing, and data FAIRification

#### Tick specimen metadata and processing

##### Field samples

Six questing *I. ricinus* adult females, Ir_f1, Ir_f3, Ir_f6, Ir_f11, Ir_f12, and Ir_f16, were collected by blanket dragging in a planted forest region (f) in the Netherlands. Another eight adult females were collected in a coastal sand dune coastal area (d) in the Netherlands and labeled as Ir_d1, Ir_d2, Ir_d3, Ir_d4, Ir_d5, Ir_d6, Ir_d7, and Ir_d9. The exact locations are in Table 1. Ticks were kept at −80°C until further processing.

**TABLE 1:** Origin and deep sequencing of adult *I. ricinus* ticks

Description	Collection date	Location (habitat type)	Name	ONT (Gb)	Illumina (Gb)
Questing adult female	14 October 2021	52.3037; 4.5331 (coastal dune)	Ir_d1	65.7	157.0
			Ir_d2	19.4	172.4
			Ir_d3	16.4	91.4
			Ir_d4	14.4	156.2
			Ir_d5	14.9	115.8
			Ir_d6	32.9	192.0
			Ir_d7	48.3	169.9
			Ir_d9	38.4	158.5
Questing adult female	23 March 2022	51.9769; 5.6992 (planted forest)	Ir_f1	144.1	137.7
			Ir_f3	88.4	105.5
			Ir_f6	69.7	111.3
			Ir_f11	63.7	140.6
			Ir_f12	39.0	120.0
			Ir_f16	67.1	139.1
Questing nymph lab-reared to female	9 January 2020	51.5839; 5.4159 (planted forest)	F1	53.0	129.8
			F2	94.8	104.8
*R. helvetica* culture from questing male	14 August 2018	52.4347; 4.6258 (coastal dune)	DK2	1.22	0.63
*R. helvetica* culture from questing nymph	2 July 2013	48.3177; 12.5390 (planted forest)	OB144/2013	8.71	4.55

##### Lab-reared adult females

Questing *I. ricinus* nymphs were collected by blanket dragging and morphologically identified to species level using an identification key (44). Nymphs were fed on an artificial membrane blood-feeding system, as described in reference 45. Two lab-reared adult females, F1 and F2, were used in this study (Table 1).

### DNA isolation and sequencing of biospecimens

DNA extraction and sequencing were performed at Future Genomics Technologies BV (Leiden, The Netherlands). Ticks were ground to a fine powder using a pestle and mortar with liquid nitrogen. To extract high molecular weight DNA, a Genomic-tip 20 /G kit (Qiagen Benelux BV, Venlo, The Netherlands) was used as per protocol.

DNA quality was measured via electrophoresis in Genomic DNA ScreenTape on an Agilent 4200 TapeStation System (Agilent Technologies Netherlands BV, Amstelveen, The Netherlands) and total DNA using a Qubit 3.0 Fluorometer (Life Technologies Europe BV, Bleiswijk, The Netherlands). Prior to sequencing, detection of *R. helvetica* was done by qPCR of the *gltA* gene using Rick_HelvgltA_F2: ATGATCCGTTTAGGTTAATAGGCTTCGGTC and Rick_HelvgltA_R2: TTGTAAGAGCGGATTGTTTTCTAGCTGTC as forward and reverse primers as described (46) (File S1.1).

One microgram of the DNA samples was used to prepare a 1D ligation library using the Ligation Sequencing Kit (SQK-LSK110) as per protocol (Oxford Nanopore Technologies [ONT], Oxford, United Kingdom). ONT libraries were tested on a MinION flowcell (FLO-MIN106) and subsequently run on a PromethION flowcell (FLO-PRO002) using the following settings: basecall model: high-accuracy; basecaller version: Guppy 5.0.17. Parallel aliquots of the DNA samples used for ONT sequencing were used to prepare Illumina libraries using the Nextera DNA Flex Library Prep Kit as per protocol (Illumina Inc., San Diego, CA, USA). Library quality was measured using an Agilent 4200 TapeStation System as described above. Genomic paired-end (PE) 150 nt libraries were sequenced using the Illumina NovaSeq 6000.

### DNA isolation and sequencing of *R. helvetica* strains from Vero cells

*R. helvetica* strain OB144 originated from a questing nymph in a planted forest in Germany, and DK2 from a questing adult male tick (Table 1). Strains were cultured and harvested from VERO cells as described (47, 48) with two modifications: here, the needle and syringe protocol was implemented using 26-gauge needles and a 0.45-µm syringe-driven membrane filter. Genomic DNA was extracted using the ZymoBIOMICS 96 MagBead DNA Kit (Zymo Research, Orange, CA). OB144 was sequenced by Future Genomics Technologies BV as described above. DK2 was sequenced by Baseclear (Leiden, the Netherlands) using a NovaSeq 6000 system (Illumina, San Diego, CA, USA). DK2 long reads were generated by RIVM using a Nanopore GridION sequencer using the sequencing kit LSK110. Base calling was performed using the ONT Guppy barcoding software version 4.4.1+1c81d62.

### Taxonomic classification of Illumina reads

Illumina reads were filtered with fastp v0.2.23 with parameters –correction, –disable_trim_poly_g, –length_required 50, –qualified_quality_phred 20, –unqualified_percent_limit 20. For taxonomic classification of the Illumina reads, the filtered reads were run through Kraken2 v2.1.2 in paired-end mode (–paired-end) with a confidence threshold (–confidence) of 0.5. For the Kraken2 index, the full NCBI nucleotide (nt) database (downloaded from https://genome-idx.s3.amazonaws.com/kraken/k2_nt_20230502.tar.gz) was used. The index was constructed using default parameters: kmer length of 35, minimizer length of 31, and minimizer spacing of 7. For species abundance estimation, Bracken v2.8 was used with the previously described NCBI nt kraken2 database with a read length (-r) of 150 and a threshold (-t) of 1,000 reads and level (-l) “S” for species. Eukaryotic species were filtered from the Bracken output files with the “filter_bracken_out.py” script from KrakenTools v1.2e. To get read counts below 1,000 for the resulting species, Bracken was run on the same parameters except with a threshold (-t) of 10 reads.

### Genome assemblies and annotation

A standardized hybrid workflow for metagenomics assembly (49) was used, which is registered at the WorkflowHub (50). Illumina and nanopore reads were inspected for quality using FastQC v0.12.1 (51). Illumina reads were filtered with fastp v0.23.2 (52) with parameters –correction, –dedup, –disable_trim_poly_g –length_required 50, –qualified_quality_phred 20, –unqualified_percent_limit 20. Possible Illumina spike in PhiX genomic reads contamination was filtered with BBMap/bbduk v38.95 (53) -k 31, reference (NC_001422.1). To filter Illumina reads for selected species, BBMap v38.95 (53) was used with default settings and bloom = t. Nanopore reads were filtered using minimap2 v2.24 (54) default settings, –x and map-ont. Mapped reads were kept. Genome sequences used for filtering were extracted from the European Nucleotide Archive. For *Rickettsia,* all available *Rickettsia* reference genomes were used. For *M. mitochondrii,* the reference genome was CP002130.1. For mitochondrial reads, all available *Ixodes* mitochondrial genomes were used. A list of genome accessions for filtering can be found in File S1.2.

### *R. helvetica*: *rickA* gene variability analyses

Questing nymphs were collected in Duin & Kruidberg, Schiermonnikoog, Gieten, Veldhoven, Montferland, and Wassenaar, the Netherlands as described (55), immersed in 70% alcohol and stored at −20°C. DNA was extracted by alkaline lysis as described (56). *R. helvetica* was detected by qPCR targeting the *gltA* as described above. In the next step, the *rickA* gene was detected by PCR assay targeting a 600 nt fragment of the gene on qPCR-positive samples, as described (8). DNA-free water was used as a negative control. Primers used were RIC1200 F2 GGCAAAATGTTAAAAATGTTT and RIC + 430 R3 CCRGYTTTTTAACCGTAGTAG. The *rickA* gene PCR products and the sequences from the new *R. helvetica* genomes were aligned with ClustalW using Geneious 11.1.4 software (57, 58).

### Phylogenetic analysis of *Ixodes ricinus* mitogenomic data

ClustalW alignments were computed using Geneious 11.1.4 software (57, 58). Phylogeny was inferred by IQTREE 2.1.3 (59), and the best-fit evolution model was selected based on the Bayesian information criterion (BIC) computed by implemented ModelFinder (60). Branch supports were assessed by the ultrafast bootstrap (UFBoot) approximation (61) and the Shimodaira–Hasegawa-like approximate likelihood ratio test (SH-aLRT) (62). Tree was rooted at the midpoint. No out-group was included. The tree was visualized and edited in FigTree (v1.4.1) and Inkscape (v 0.91) (63).

### Cophylogenetic analysis

The single-copy core genes of 11 *R*. *helvetica* and 12 *M*. *mitochondrii* genomes were identified in Anvi’o 7 and made up of 1,484 and 1,127 single-copy gene (SCG) clusters, respectively. Gene alignments from SCG were extracted and concatenated using the program anvi-get-sequences-for-gene-clusters, with parameters: –concatenategene-cluster; –report-DNA-sequences. Maximum likelihood phylogenetic trees were constructed in IQ-TREE v2.2.0 (59). The best model according to the Bayesian information criterion (BIC) was selected with Model Finder (60) as implemented in IQ-TREE. All models were run with ultrafast bootstrapping (61). eMPRess, a program for phylogenetic tree reconciliation under the duplication-transfer-loss (DTL) model (64), was used to determine whether the mitochondrial genome of *I. ricinus* and *I. ricinus* symbionts: *M. mitochondrii* and *R. helvetica* coevolved by comparing their phylogenetic relationships. The input trees were based on the results of phylogenetic analyses. The analysis was conducted using the default eMPRess parameters.

### Genome-scale metabolic model reconstruction and analysis

Genome-scale metabolic models (GEMs) were developed using CarveMe (v1.5.1) (65), starting with their annotated genome sequences (Ir_d9Mm and Ir_d9Rh). For reaction essentiality analysis, we performed gene or reaction deletion analysis using flux balance analysis (FBA) (File S2). MEMOTE (v0.13.0) (66) was used to assess the quality of the GEMs. In addition, a full reaction essentiality analysis was performed on both GEMs, identifying all metabolites for which the symbionts rely on the host (File S2). Furthermore, we performed a permutation test to assess the significance of the overlap in essential reactions between the symbionts. COBRApy (v0.26.3) (67), employing Python (v3.11.3), was used for modeling simulation and analysis of each GEM using the IBM CPLEX Solver. Scripts used to reproduce the model simulations are provided on GitLab (https://git.wur.nl/unlock/projects/ticks).

### Immunofluorescence localization studies of *R. helvetica* in ticks

Fifteen *I. ricinus* questing female ticks were collected in the Amsterdamse Waterleidingduinen, the Netherlands, and morphologically identified to species level using an identification key (44). All ticks were kept alive until further processing. Ticks were dissected, and the gut, Malpighian tubules, salivary glands, reproductive system, and synganglion were collected. Tick carcasses were screened for the presence of *R. helvetica* DNA by qPCR. The same was performed on 10 control ticks from a laboratory colony (BIPAR, France).

Organs were fixated in 4% paraformaldehyde (PFA) in PBS for 2 h and then replaced with 70% ethanol and stored at −20C until used. The organs were washed with 0.5% Triton X-100 in PBS (PBST) five times, then replaced by 5% normal goat serum (Sigma) in PBST and incubated for 1 h at room temperature. Afterward, the serum was replaced by *R. conorii* commercial antibody 1:30 in PBST and incubated at 4°C for 2 days. Samples were incubated with DAPI before mounting in Prolong Antifade Diamond Mountant (Life Technologies), and images were obtained using a z-stack tool in Leica DMI8 confocal microscopy. Assembled z-stacks were adjusted in Adobe Photoshop version 24.0.1 (Adobe Inc.).

### Statistical analysis

Haplotype diversity (Hd), variance of Hd, nucleotide diversity (*π*), variable (polymorphic) sites, and parsimony informative sites were calculated for mitochondrial genomes of *I. ricinus* using the DNA SP6 (v6.12.03) (68).

To test whether the composition of repeats differs per prevalence and per area type (dune vs. forest), we performed PERMANOVA based on Bray-Curtis dissimilarity matrix using the adonis2 function from the vegan package (69).

## RESULTS

### Deep sequencing of *I. ricinus* adult females reveals the presence and diversity of symbiotic bacteria

**Fig 1:**
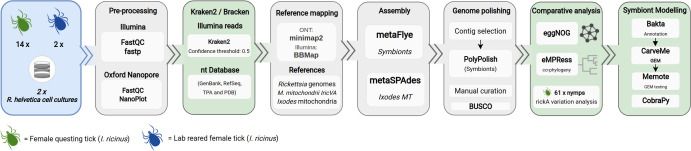
*I. ricinus* endosymbiont sequence classification, genome assembly, and analysis. Illumina reads were used as input for the Kraken2-Bracken workflow (57, 72) to obtain microbiome-related abundances and taxonomic classifications. Subsequently, a standardized workflow for hybrid metagenomics assembly was used to obtain *Rickettsia* and *Midichloria* genomes from each positive specimen (see Materials and Methods for details). Two sources of genomic DNA were used as input: *I. ricinus* metagenomic DNA obtained from adult female ticks and *R. helvetica* genomic DNA isolated from infected Vero cell lines.

Sixteen adult female specimens were sequenced using both Illumina and ONT (Oxford Nanopore Technologies) methodologies: eight from a coastal sand dune area (d), six from a planted forest area, and two specimens molted under laboratory conditions. Using Illumina 150 × 150 nt paired-end reads as input and the nt database (70) as a reference, we applied a Kraken2-Bracken workflow (46, 71) to estimate for each specimen the abundances and taxonomic classifications of the microbiome-related Illumina reads obtained (Fig. 1, step 1). In total, more than 90% of the read pairs that were attributed to the bacterial domain could be classified at the species level.

**Fig 2:**
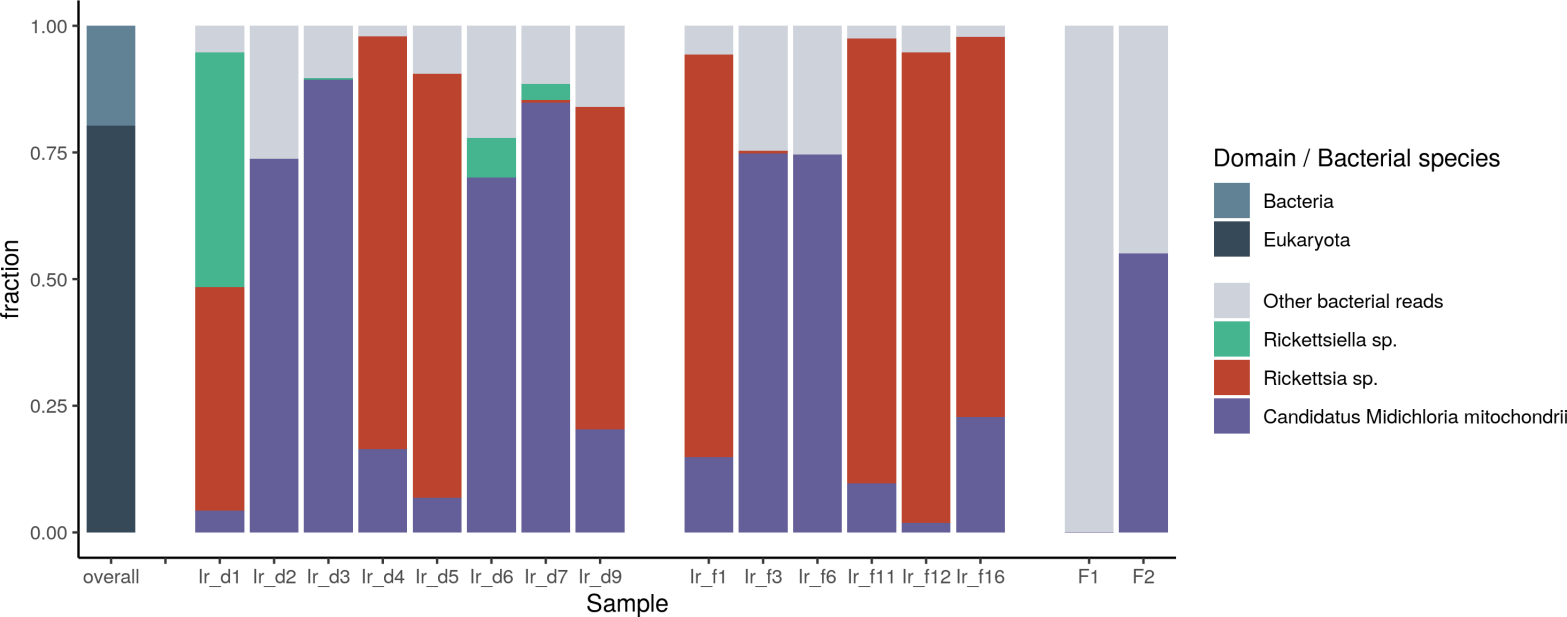
Kraken2-Bracken distribution of classified Illumina paired-end reads at the species level. Default parameters for kmer length (35), minimizer length (31), and minimizer spacing (7) were used in this analysis. Left: top-level classification, right: classification of the bacterial domain at species level. The top three most prevalent species are indicated. A more detailed analysis is available in File S1.3.

A significant number of paired-end reads (with cumulative scores of >20,000 read pairs) were assigned to known tick-borne pathogens: *Anaplasma phagocytophilum* was present in tick Ir_d1 and Ir_d9; *Borrelia afzelii* was present in tick Ir_d9; and *Neoehrlichia mikurensis* (73) in tick Ir_f3. *Rickettsiella* species have recently been identified as facultative endosymbionts of *I. ricinus* ticks (74), and with more than 1 million read pairs assigned, *Rickettsiella* species appear to be present in Ir_d1, Ir_d6, and Ir_d7 (Fig. 2). *Rickettsia* was detected in eight samples, and in almost all cases, *R. asiatica* was the most prominent classified species, followed by *R. helvetica* and *R. conorii* (File S1.3).

### Genome reconstruction of *M. mitochondrii* endosymbiotic strains

Kraken analysis revealed that 15 ticks are positive for *M. mitochondria* (File S1.3). The exception was F1, raised in a laboratory setting (Fig. 2). High-quality MAGs of *M. mitochondrii* could be obtained from 11 ticks and assembly drafts from the other four (File S1.4).

The MAGs shared over 99% ANI with the *M. mitochondrii* reference strain (75) and were characterized by a high BUSCO score (>92.9%). Genome sizes ranged from 1,190,312 to 1,191,611 bp except for the assembly obtained from tick Ir_d2, which was 1,268,036 bp due to a duplication event (File S1.3 and S1.4). Overall coverage was between 24.6 and 103.10 (File S1.10). The average guanine-cytosine (GC) content was 36.6%. All *M. mitochondrii* genomes contained three rRNAs, between 35 and 39 tRNAs, and the number of protein-coding genes varied from 1,322 to 1,448. The overall coverage of these regions was analyzed, and no deviations were observed, indicating that an assembly error is unlikely.

**Fig 3:**
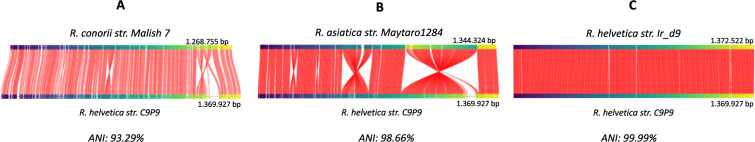
Major intragenomic rearrangements between *R. helvetica* and *R. asiatica*. FastANI orthologous mappings were done using a 3 kb fragment size. Red line segments denote the orthologous mappings between these fragments. (**A**) ANI computed between *R. conorii* strain (NC_003103.1) as query and *R. helvetica* strain C9P9 (NZ_CM001467.1) as reference. (**B**) ANI computed between *R. asiatica* strain (NZ_AP019563) as query and *R. helvetica* strain C9P9 as reference. (**C**) ANI computed between the *de novo* assembled *Rickettsia* strain from tick Ir_dr9 as query and *R. helvetica* strain C9P9 as reference.

Among the eleven *de novo Midichloria* genome assemblies, a high level of genomic collinearity was observed (Fig. 3, panel B, File S3); however, in comparison with the reference strain, a significant amount of structural variation was observed. Genome variant calling revealed strain variations. The total number of variants ranged from 604 to 964 (8,509 total). Of these, 5,029 single-nucleotide polymorphisms (SNPs), 2,246 insertions, 1,989 deletions, and 335 complex variants were detected (Table 2). Although these variants likely reflect biological differences, the potential influence of (454) sequencing artifacts on insertion and deletion counts cannot be fully excluded from the interpretation.

**TABLE 2:** Genetic variation observed in 11 genomes of *M. mitochondrii* and 8 genomes of *R. helvetica* analyzed in this study

Strain	Complex^*a*^	Deletions	Insertions	SNPs	Variant total^*b*^	Genome size	% difference
*M. mitochondrii*							
F2	25	170	92	317	604	1,190,761	0.0059
Ir_d2	29	186	112	606	934	1,268,036	0.0664
Ir_d3	38	186	117	570	911	1,191,611	0.0066
Ir_d4	35	187	112	625	962	1,190,364	0.0055
Ir_d7	30	183	102	362	680	1,190,694	0.0058
Ir_d9	36	185	98	368	687	1,190,746	0.0058
Ir_f1	26	173	95	340	635	1,190,725	0.0058
Ir_f3	22	170	92	317	601	1,190,732	0.0058
Ir_f6	33	181	110	559	883	1,190,835	0.0059
Ir_f11	28	177	100	343	648	1,190,674	0.0058
Irf_16	33	191	116	622	964	1,190,312	0.0055
*R. helvetica*							
Ir_d1	3	2	43	17	65	1,372,533	0.0018
lr_d4	2	2	43	15	63	1,372,384	0.0017
Ir_d5	2	2	41	19	64	1,372,519	0.0018
Ir_d9	3	2	43	18	66	1,372,522	0.0018
Ir_f1	2	3	44	21	71	1,372,458	0.0018
Ir_f11	2	2	43	13	60	1,372,442	0.0018
Ir_f12	2	2	43	21	69	1,372,202	0.0016
Ir_f16	2	4	44	31	82	1,372,575	0.0019

^*a*^ Complex: the number of multiple nucleotide polymorphisms.

^*b*^ Variant total: the total amount of variants between the newly obtained and reference genome.

EggNOG-based functional analysis suggests that the reference genome, and the 11 newly sequenced genomes encode a (near) complete folate biosynthetic pathway (six of the seven required genes detected) and a likely complete biotin biosynthetic pathway (all required genes detected). COG0331 is present in all newly sequenced genomes but absent from the reference genome. COG0331 corresponds to malonyl CoA-acyl carrier protein transacylase, encoded by the *fadD* gene, and is responsible for initiating the type II fatty acid synthesis pathway (76). A complete eggNOG-based functional analysis is provided in File S1.5.

### Genome reconstruction of *R. helvetica* endosymbiotic strains

Of the 14 specimens collected in the field (Table 1), eight tested positive for the presence of *R. helvetica* DNA by qPCR analysis (File S1.1). Kraken analysis using the complete NT-database as a reference, however, suggested that *R. asiatica*, *R. conorii*, and *R. helvetica*, three species that share almost identical genome sequences, appeared to exhibit a significant concurrent presence in these ticks (File S1.9). We used FastANI (77) to gain more insight into their evolutionary distances. A commonly used operational definition for prokaryotes states that if two genomes have an average nucleotide identity (ANI) of over 95%, they belong to the same species. Between them, the reference strains of *R. helvetica* and *R. asiatica* show >98.66% ANI and in cross-comparison with *R. conorii,* >93.3% ANI. Although these high identity scores explain the Kraken results, it is important to note that FastANI revealed significant structural variation (Fig. 3). An ANI analysis between the reference strain *R. helvetica* and the reconstructed genomes showed an ANI value >99.9% and >98.6% for *R. asiatica,* which, according to the rickettsial classification guidelines (OrthoANI > 99.19%), indicate a clear species association toward *R. helvetica* (78).

From the eight positive specimens, we reconstructed a complete genome representation of *R. helvetica*. Additionally, two genomes (DK2 and OB144) were obtained using Vero cell lines. The 10 *de novo* assemblies exhibited high completeness, with BUSCO scores exceeding 98.9%. Genome sizes ranged from 1,372,202 bp to 1,372,575 bp, approximately 2.2–2.5 kb larger than the C9P9 reference genome (1,369,927 bp). Overall coverage ranged from 37,40× to 493,90× (File S1.10). All new *R. helvetica* genomes contained 3 rRNAs, 33 tRNAs, and between 1,630 and 1,664 protein-coding genes per assembly, including a complete set of genes for *de novo* folate biosynthesis (File S1.4). The overall coverage of these regions was analyzed, and no deviations were observed, indicating that an assembly error is unlikely.

Consistent with the reference strain, all assemblies included a plasmid of 47184–48,139 bp, encoding 56–59 proteins, including 39–40 hypothetical proteins. Plasmid association was verified using the reference strain and the DK2 and OB144 strains, all of which were obtained from culturing in Vero cell lines. Overall ANI values between the strains, including the plasmids, were on average 99.9% (File S1.12). No plasmid-specific reads were detected in the *R. helvetica*-negative specimens, indicating an association between *R. helvetica* and the plasmids identified.

Genome variant calling among the eight *I. ricinus*-derived genome assemblies revealed a total number of variants ranging from 63 to 82 (540 in total). Of these, 155 single nucleotide polymorphisms (SNPs), 344 insertions, 19 deletions, and 18 complex variants were detected (Table 2).

Overall, the new (meta)genome assemblies revealed a very high level of genomic collinearity, among themselves, and with the *R. helvetica* reference strain (Fig. 3 panel C). Most variation is due to what appears to be gene-splitting events, leading to two or more open reading frames. Between the eight genome assemblies that were directly obtained from the *I. ricinus* specimens, we found three such gene-splitting events. In contrast, we observed a notable increase of such events in the genomes of the DK2 strain and the C9P9 reference strain both obtained using infected Vero cells (File S1.6).

Due to such gene splitting events, the DK2 genome encodes a non-functional RickA protein (File S1.7). RickA protein plays a role in actin-based cell-to-cell motility, and *rick*A is therefore considered to be a putative virulence gene (34, 79). Further inspection of the new *R. helvetica* genomes revealed high genetic variability in the *rickA* gene due to copy number variation of a 33-nt-long repeat motif encoding 11 amino acids including five adjacent prolines ranging in number from 5 (Ir_d4) to 13 (OB144).

To gain more insight into this genetic variability, we sequenced the fragment of the *rick*A gene containing the repeat motif from 61 questing nymphs gathered from various forest and dune regions (File S1). The results confirmed the observed copy number variation, which varied from 5 to 11. No significant compositional difference was observed between dune and forest areas (F 1,7 = 1.2, *P* = 0.3772), as well as between eight sampled locations with varying *R. helvetica* prevalence (F 1,7 = 0.4, *P* = 0.9101).

### Genome-scale metabolic modeling of *M. mitochondrii* and *R. helvetica* reveals metabolic dependencies

Due to their adaptation to rely on the host organism for survival, *M. mitochondrii* and *R. helvetica* have reduced genomes encoding reduced metabolic networks. The automatically generated GEM of *M. mitochondrii* (Ir_d9_Mm) is comprised of 1,126 reactions, 881 metabolites, and 266 genes, whereas the GEM of *R. helvetica* (Ir_d9_Rh) consists of 1,123 reactions, 851 metabolites, and 274 genes (Table 3).

**TABLE 3:** Summary of genome-scale metabolic models of *M. mitochondrii* and *R. helvetica*

Parameter		Ir_d9_Mm	Ir_d9_Rh
Total no. of reactions		1126	1123
Total no. of metabolites		881	851
Total no. of genes		266	274
Reactions			
Transport		349	399
Metabolic		630	581
Without GPR associations		337	353
Metabolites			
Unique metabolites		881	851
Medium components		144	139
Genes: enzyme complexes		36	49

**Fig 4:**
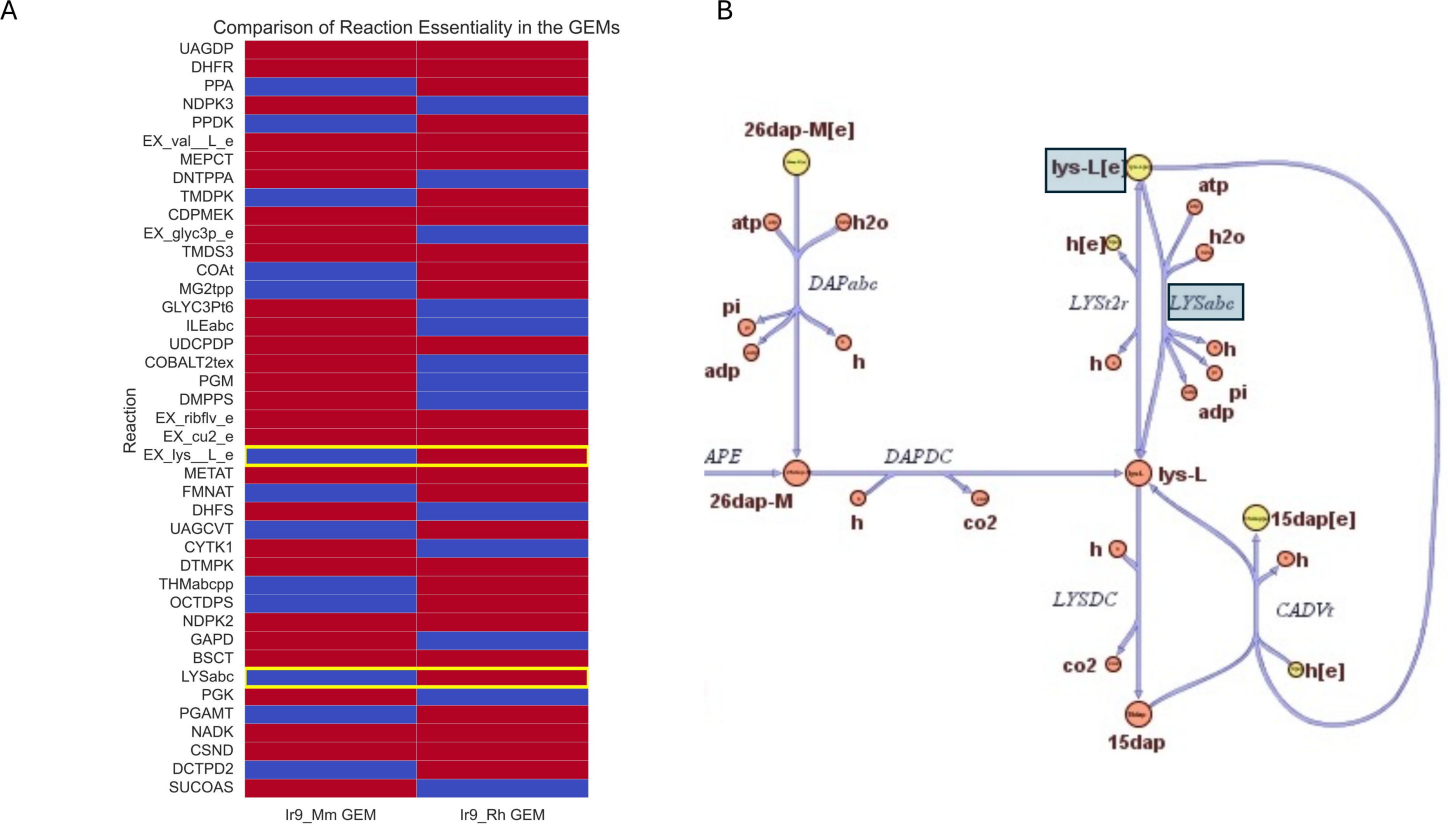
Genome-scale metabolic constructions and analysis of *M. mitochondrii* and *R. helvetica*. Heatmap comparing *M. mitochondrii* (Ir_d9_Mm GEM) and *R. helvetica* (Ir9_Rh GEM) where a subset of the essential reactions is depicted. Furthermore, the blue and red panels represent which reactions are not essential or essential, respectively (A). Note: complete heatmap comparing the reaction essentiality of the two GEMs is contained in the supplementary material. Network map showing the network map of lysine synthesis pathway highlighting specific essential reactions in the Ir_d9_Mm GEM for *M. mitochondrii* (Ir_d9_Mm GEM) (B).

MEMOTE analysis (66) revealed that both GEMs achieved high-quality overall scores of 84% (Ir_d9_Mm) and 68% (Ir_d9_Rh), respectively. (File S2). The network motifs of the two models point to differences in central carbon and fatty acid metabolisms. For instance, the central carbon metabolism appears to be more tightly connected to nitrogen metabolism in the Ir_d9_Rh GEM compared with Ir_d9_Mm GEM. Despite these differences, the two GEMs share 802 reactions. Each GEM also contained unique reactions and pathways: 300 for Ir_d9_Rh GEM and 429 for Ir_d9_Mm GEM. More specifically, the Ir_d9_Mm GEM contained unique reactions and pathways related to pantothenic acid (vitamin B5) and niacin (vitamin B3). Reaction essentiality analysis indicates that 8.70% and 8.17% of the reactions in *M. mitochondrii* and *R. helvetica*, respectively, are essential for *in silico* growth (File S2) and suggests that reactions linked to lysine synthesis pathways are vital for the growth of *R. helvetica* but not for *M. mitochondrii* (Fig. 4). Our analysis revealed that in addition to lysine, *R. helvetica* depends on the host for several amino acids such as valine, tyrosine, and isoleucine, reflecting its extensive auxotrophy (File S2).

### Cophylogenetic analysis between *I. ricinus* and symbionts

The phylogenetic analysis of the mitogenomic data generated for 16 *I. ricinus* ticks was performed, including 33 mitochondrial genomes of *I. ricinus* obtained from the public databases. The results suggest a lack of a locality-dependent structure based on habitat type (dune and forest) from where ticks were collected (File S4; Fig. S4.1). The phylogenetic tree, however, did show an evolutionary distance between the different reconstructed mitochondrial genomes from the different samples.

**Fig 5:**
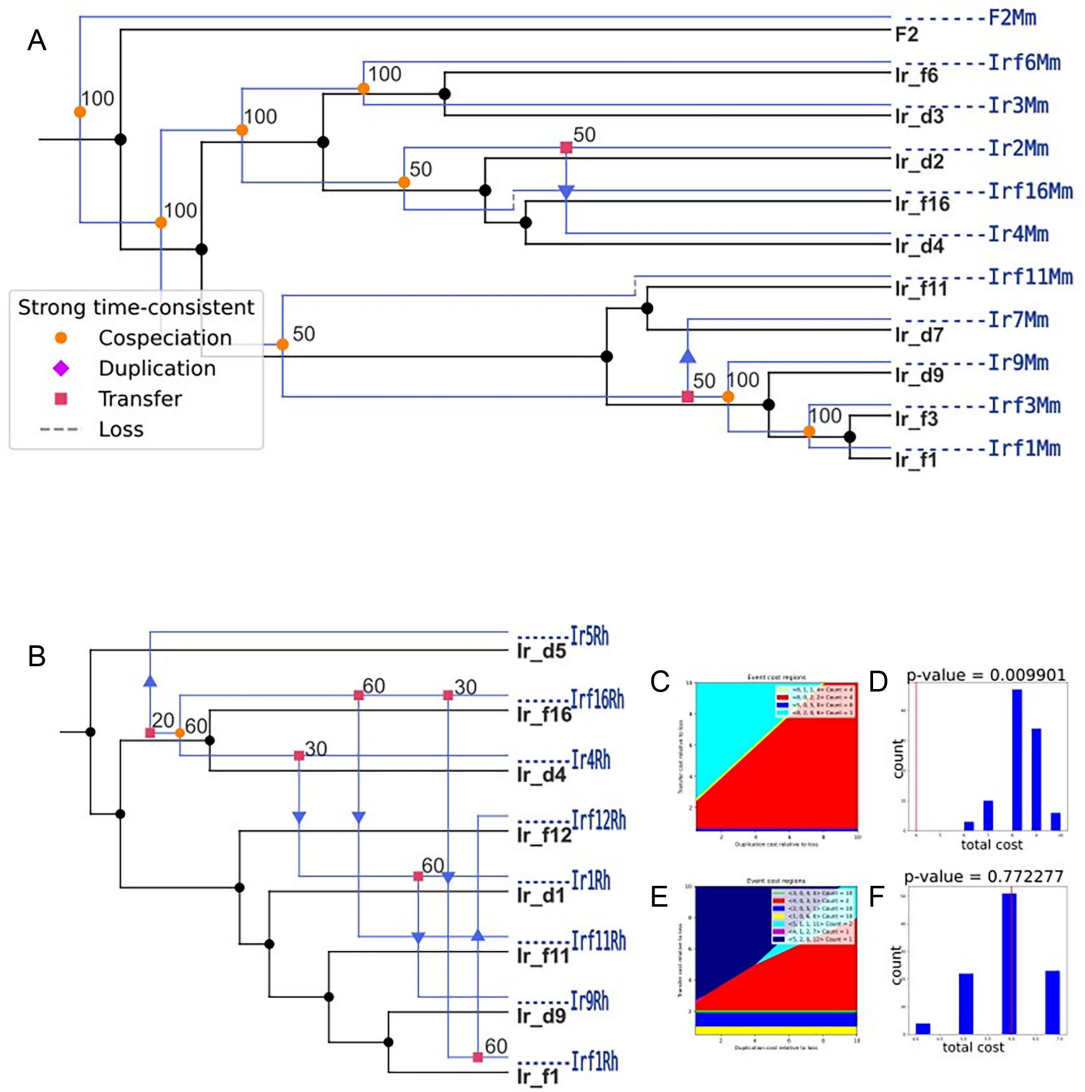
Cophylogenetic analysis between *I. ricinus* ticks and its symbionts. (**A**) Phylogenetic reconciliation of *I. ricinus* (displayed in black) and *M. mitochondrii* (in blue). (**B**) Phylogenetic reconciliation of *I. ricinus* (displayed in black) and *R. helvetica* (in blue); (**C and D**) *P*-value histogram of host and *M. mitochondrii*; (**E and F**) *P*-value histogram of host and *R. helvetica*; the optimal reconciliation cost of the coevolution trees is indicated with a red line, and the optimal cost of the same trees constructed with tip associations permuted at random is shown in blue columns.

To determine whether symbiont-tick pairs are co-evolved, the eMPRess phylogenetic tree alignment software, following the duplication-transfer-loss model, was used to compare the phylogenetic relationships of symbionts and ticks. This analysis was preceded by the reconstruction of phylogenetic trees for *M. mitochondrii* and *R. helvetica* genomes obtained in this study (see File S4; Fig. S4.1 and S4.2). The reconciliation analysis was performed separately for two pairs: *M. mitochondrii–I. ricinus* and *R. helvetica–I. ricinus* (Fig. 5).

The results showed that a total of 12 events, including eight cospeciation events, two transfers, and two losses, occurred between *M. mitochondrii* and *I. ricinus* over evolutionary time; of these events, cospeciation was robustly supported in six out of eight events. The results of this analysis refuted the null hypothesis that the tick and *M. mitochondrii* trees and tip associations are formed due to chance at the 0.01 level in host-symbiont relationships (*P*-value = 0.0099). Therefore, we concluded that these *I. ricinus–M. mitochondrii* pairs have coevolved. The results for the *R. helvetica–I. ricinus* pair suggest rejection of the coevolution hypothesis (*P*-value = 0.77).

### *Rickettsia helvetica* localization in adult female ticks

**Fig 6:**
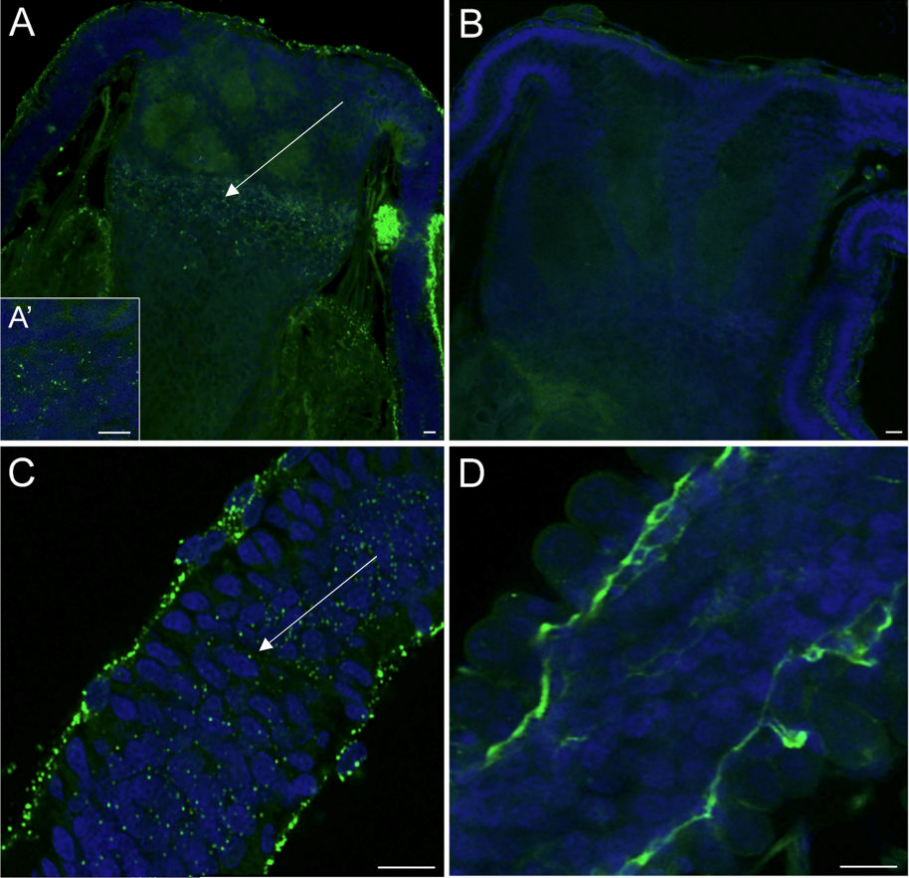
Localization of *R. helvetica* in adult females. Blue: DAPI, green dots: *Rickettsia*-specific binding. (**A**) *R. helvetica*-positive *receptaculum seminis* X40 A’) *R. helvetica*-positive *receptaculum seminis* ×100, (**B**) control *receptaculum seminis,* (**C**) *R. helvetica*-positive oviduct X40, and (**D**) control oviduct. Note that tissue-specific autofluorescence can be observed in the treatment and control groups. Scale bar: 10 µm

The distribution of *R. helvetica* in adult female ticks was studied to examine the nature and uniformity of its presence in tick tissues and to identify possible transmission routes. *R. helvetica* was successfully visualized using laser microscopy in five out of the 15 females (Fig. 6). These ticks were also qPCR positive for *R. helvetica*. All controls were negative (File S1.8). *R. helvetica* was observed in salivary glands, Malpighian tubules, the gut, and reproductive organs (File S5). The distribution of *R. helvetica* in tick organs varied between specimens. In the reproductive system, *R. helvetica* was observed in the female oviduct (two ticks, Fig. 6C and D) and in the *receptaculum seminis* (RS) (three ticks, Fig. 6A and B), which acts as the storage site for sperm introduced during copulation.

## DISCUSSION

### Endosymbiont genome sequencing

This study explores the transmission dynamics and lifestyle of symbionts associated with *I. ricinus*. Although traditional approaches for whole-genome sequencing of obligate intracellular bacteria have often relied on isolation and host-based propagation, recent advances have enabled direct sequencing without these steps (80, 81). In this study, we applied a high-depth, long-read in combination with a short-read direct deep-sequencing strategy to achieve the closed assembly of a relatively large set of samples. This approach allows for a more efficient reconstruction of the *M. mitochondrii* and *R. helvetica* genomes, providing insights into their transmission dynamics and lifestyle.

In this study, *M. mitochondrii* was detected in 15 specimens, except for a single specimen molted under laboratory conditions. This absence aligns with previous observations that both the prevalence and abundance of *M. mitochondrii* decrease in laboratory-maintained tick colonies (82–84). Such reductions suggest that the bacterium plays a role specific to natural environments, potentially linked to host interactions, environmental pressures, or nutrient availability absent in lab settings.

Previous research indicates that laboratory interventions can significantly diminish *M. mitochondrii* populations. For instance, a study by Guizzo et al. demonstrated that administering tetracycline to *I. ricinus* ticks via an *ex vivo* membrane feeding system significantly reduced the abundance of *M. mitochondrii* in their ovaries (82). This reduction persisted across subsequent generations, leading to a complete elimination of the symbiont. Similarly, Ninio et al. observed that antibiotic treatment of *I. ricinus* resulted in a decreased load of *M. mitochondrii* following a blood meal (85). These findings suggest that laboratory interventions, such as antibiotic administration, can effectively reduce or eliminate *M. mitochondrii* in tick populations.

In addition to *M. mitochondrii*, *R. helvetica* was present in eight specimens, whereas *Rickettsiella* species were prominently present in three specimens. A high overall abundance of *Pseudomonas*, *Mycobacterium*, and *Mycobacteroides* species was also observed. It is important to note that the sensitivity of a Kraken2 analysis is significantly influenced by the type of genetic data available in the nt database—whether 16S sequences, individual gene fragments, or complete genomes—indicating that Kraken read counts cannot be directly used to estimate relative species abundances.

As there are reference genomes available for *M. mitochondrii* and *R. helvetica*, employing fixed-length Illumina read pairs as input allowed us to directly utilize the Kraken2 output for estimating whether there exists adequate short-read genome coverage to reconstruct the complete microbial genomes of interest. In alignment with the outcomes from Kraken2, we were able to obtain 11 complete and four draft genomes of *M. mitochondrii* and, coinciding with qPCR analysis results, eight complete *R. helvetica* genomes along with their associated plasmids.

### M. mitochondrii

The genomes of *M. mitochondrii* as presented in this study are approximately 5% larger and have a structural arrangement that differs from the published assembly (accession no.: NC_015722). The observed differences in genome size and structure could be due to the use of different technological platforms, assembly methods, and geographic origin (the Netherlands vs. Italy). The extraction of 11 complete *M. mitochondrii* genomes, each from a single tick, provides insight into the genomic potential and structural variability of this symbiotic bacterium in its tick host.

*M. mitochondrii* exhibits a unique feature to colonize multiple compartments in a tick body, including ovaries, salivary glands (86), Malpighian tubules, tracheae, and guts (13). In this context, each genome generated in our study represents a mixture of genomes from bacteria inhabiting different ecological niches within the tick body. Subsequently, in the eMPRess reconciliation analysis, we showed a significant coevolutionary relationship between *M. mitochondrii* and its tick host. This finding supports the notion of a long-standing symbiotic relationship between *M. mitochondrii* and *I. ricinus*, suggesting that vertical transmission is likely the primary mode of symbiont transfer.

The strong cophylogeny observed between *M. mitochondrii* and its host’s mitochondria in this study further supports the idea that vertical transmission is the dominant mode of inheritance, opposing the hypothesis of horizontal transmission.

ANI analysis of the *M. mitochondrii* complete draft genomes revealed two main groups of genome arrangements: one consisting of *M. mitochondrii* isolated from Ir_*f1, Ir_d3, Ir_d7, Ir_f3,* and *Ir_f11*, and another consisting of *Ir_d2, Ir_d4,* and *Ir_f16* (File S1.12). This grouping further supported the strong cophylogeny observed between *M. mitochondrii* and its host’s mitochondria (File S4.1).

Although *M. mitochondrii* has been detected in tracheae, salivary glands, and rostrum, as well as in the blood of experimentally infested rabbits, horizontal transmission is unlikely to play a major role in its spread within natural tick populations. However, given the localized scope of this data set, broader conclusions regarding the absence of horizontal transmission would require further investigation across diverse populations and environments.

### R. helvetica

The prevalence of *R. helvetica* in questing ticks has been shown to greatly differ between dune and forest areas in the Netherlands (27, 33, 87). Although our findings point to some heterogeneity in the *rickA* virulence gene, there seems to be no genetic basis for the differences in the prevalence of *R. helvetica* in dune versus forest areas. Thus, two remaining possibilities should be further explored. First, the possibility of *R. helvetica* giving ticks in dune areas an adaptive advantage in relation to local abiotic conditions has not been fully explored. The role of *R. helvetica* in this case could be related to nutrient acquisition, waste elimination, or physical characteristics. For example, *Rickettsia* has been shown to enhance tick motility in *Dermacentor variabilis* (88). Second, the possibility of interactions with other symbionts within the tick host should be explored as *R. helvetica* has been shown to modulate the microbiome of *I. ricinus* (89), and it has been found to be negatively correlated with certain symbionts such as *Spiroplasma ixodetis* and *M. mitochondrii* (2, 33). It has been hypothesized that perhaps the biological roles of *S. ixodetis* and *R. helvetica* overlap, making their simultaneous maintenance energetically superfluous for the tick host (33), but this theory has yet to be proven. Nor has the option of other bacterial interactions been fully researched.

Our comparative analyses showed near absolute genetic homogeneity among the generated *R. helvetica* genomes (99.96%–100%), and only a slight deviation from the published strain C9P9. In the latter case, the observed differences in genome size could be due to different isolation and assembly methods or even geographic origin. The congruous genetic build-up among the *R. helvetica* genomes was punctuated almost exclusively by the gene encoding for the surface protein RickA. The sequence of the *rickA* gene showed high genetic variation between the genomes generated in this study as well as between genes obtained from 61 additional ticks from the Netherlands. The RickA protein has been shown to induce actin polymerization and hence actin-based motility in some *Rickettsia* species (37, 80). However, a previous study found the RickA protein in *R. helvetica* isolate AS819 to be insufficient to promote actin-based motility *in vitro*, which spread instead through cell breakdown and bacterial release (26). The inability of the studied isolate to spread through actin-based motility was explained by a disrupted *sca*2 gene, another virulence gene believed to have a more prominent role than the *rickA* gene in the cell-to-cell spread of *Rickettsias* (90). Our genomes revealed no truncated *sca2* sequences; however, the *rickA* gene greatly differed in length due to a repeat motif. We hypothesize these repeats may affect the functionality of the RickA protein within *R. helvetica*, giving rise to variation in virulence. This observation is supported by the dose-dependent manner in which RickA induced cell motility in *R. conorii* (37). Moreover, when expressing a heterologous *rickA* gene, *R. bellii* moved at higher velocities than its wild-type counterparts (91). If the diverse *rickA* sequence length translates into different motility-associated phenotypes, it could explain the seemingly sporadic pathogenicity we observe for *R. helvetica*, as well as its low and inconsistent prevalence rate in wildlife hosts. This heterogeneity could also explain why two different mice models showed no substantial *rickettsia* following an infection challenge with *R. helvetica* (92) and should be considered when exploring the infection potential of *R. helvetica* strains.

To date, no wildlife reservoir has been found for *R. helvetica*. This has led to the assumption that *I. ricinus*, which transmits *R. helvetica* transovarially (93), acts as its main reservoir in nature (27). This close tick-symbiont interaction was not reflected in our findings, which showed the absence of co-phylogeny between *R. helvetica* and the mitochondrial genomes of *I. ricinus*. The lack of co-evolution between *I. ricinus* and *R. helvetica* may be partly due to a mixed transmission strategy encompassing vertical and horizontal routes. The presence of *R. helvetica* in the RS, as shown by our immunofluorescence findings, strongly suggests the possibility of paternal transmission for *R. helvetica* in *I. ricinus*. Paternal transmission of *Rickettsia* has been identified in the leafhopper *Nephotettix cincticeps* (94), and it has been hypothesized that a mixed mode of transmission (maternal and parental) might allow for the mixing of different symbiont lineages, thus allowing for the evolution of virulent variants (95). *Rickettsia* has also been detected in the spermatids of *Dermacentor silvarum* and *I. ricinus* ticks (96, 97). In this study, adult females were studied, as pathogens are thought to be transmitted to humans mainly by nymphs and female ticks. The possibility of a different genetic makeup for *Rickettsia* populations found in reproductive tick organs should be considered in future studies in both male and female ticks.

### Symbiont metabolic integration

Genome-scale metabolic models (GEMs) provide a means to uncover new biological insights that go beyond what genomics can offer. A key application involves predicting the viability of an organism under specific conditions. This simulation approach has been utilized to identify potential metabolic drug targets, enabling the effective targeting of pathogens (98).

Due to their long-term relationship with the tick host, both *M. mitochondrii* and *R. helvetica* underwent genome reduction, leading to incomplete metabolic networks. The analysis suggests that *M. mitochondrii*’s *in silico* growth necessitates the inclusion of pantothenate (vitamin B5) and glycerol-3-phosphate (G3P) in the growth medium. G3P, a key metabolite in metabolic pathways such as glycolysis, gluconeogenesis, and lipid metabolism, is abundantly available in mitochondria. Its role is pivotal in the glycerol phosphate shuttle, facilitating the transfer of reducing equivalents to the mitochondrial matrix. Pantothenate is vital for energy metabolism and the synthesis of coenzyme A (CoA), a coenzyme integral to mitochondrial metabolic reactions. Our findings about the growth requirements of *M. mitochondrii* are in line with the evolutionary adaptation of this symbiont to reside in its host’s mitochondria. Nevertheless, as ticks cannot synthesize pantothenate, there could be potentially other bacterial symbionts producing this vitamin to be utilized by *M. mitochondrii*. The GEM for *R. helvetica* highlights a dependency on lysine for *in silico* growth, as demonstrated in Fig. 4B. This requirement likely results from an incomplete lysine biosynthesis pathway, a characteristic shared with *Rickettsia prowazekii* (99, 100), as previously identified. Research has consistently shown that *Rickettsia* species lack comprehensive amino acid biosynthesis pathways, particularly for essential amino acids such as lysine (72, 101). Therefore, *R. helvetica* must obtain lysine from the blood meal ingested by its tick host or from other tick symbionts; however, the latter has not been explored. In some cases, *Rickettsia* species have been found to carry out only the initial stages of lysine biosynthesis, leading to the production of diaminopimelate rather than lysine itself, necessitating the acquisition of lysine from their external environment (101).

Simultaneously, we examined how these symbionts could contribute to their tick host by providing essential micronutrients. All *R. helvetica* genomes harbor a complete set of genes for *de novo* folate biosynthesis, in line with previous findings (13, 102). *M. mitochondrii* encodes a (near) complete folate biosynthetic pathway and a likely complete biotin biosynthetic pathway.

### Conclusion

Ticks harbor and transmit specialized symbiotic microorganisms that are crucial for their hematophagous lifestyle and occasionally serve as causal agents of diseases in humans and animals. Although these symbionts are challenging to culture and manipulate *ex vivo*, our study shows how deep sequencing allows for the direct extraction of complete genomes from tick specimens collected from their natural habitats, providing fresh insights into their structural genome variation, host interactions, and modes of transmission.

## Data Availability

The data supporting the findings of this study have been deposited in the European Nucleotide Archive (ENA). This data set includes Oxford Nanopore Technologies (ONT) and Illumina sequencing data for multiple Ixodes ricinus specimens. The project number is PRJEB70514. ONT accession numbers areas follows: ERR12319604, ERR12319605, ERR12319607, ERR12319608, ERR12319610, ERR12319612, ERR12319614, ERR12319616, ERR12319618, ERR12319620, ERR12319623, ERR12319624, ERR12319627, ERR12319628, ERR12319631, ERR12319632, ERR12319593, ERR12319595, ERR12319597, ERR12319598, ERR12319599, ERR12319600, ERR12319602. Illumina accession numbers are as follows: ERR12319603, ERR12319606, ERR12319609, ERR12319611, ERR12319615, ERR12319617, ERR12319619, ERR12319621, ERR12319625, ERR12319626, ERR12319629, ERR12319630, ERR12319633, ERR12319622, ERR12319594, ERR12319596, ERR12319601. *Midichloria mitochondrii* accession numbers are as follows: GCA_963853845, GCA_963854075, GCA_963853965, GCA_963853945, GCA_963853935, GCA_963854045, GCA_963854055, GCA_963853925, GCA_963853985, GCA_963854105. *Rickettsia helvetica* accession numbers are as follows: GCA_963854155, GCA_963853705, GCA_963853725, GCA_963853685, GCA_963853695, GCA_963853745, GCA_963853735, GCA_963853715, GCA_963854155, GCA_963970025. The code used for the model simulations can be found at https://git.wur.nl/unlock/projects/ticks, with supplemental text and figures at https://git.wur.nl/unlock/projects/ticks/-/tree/main/supplementary_material. The GEMs as well as accompanying MEMOTE (56) and FROG analysis (11) reports can be found in a public repository on BioModels: https://www.ebi.ac.uk/biomodels/. For the *M. mitochondrii* GEM, see here: MODEL2404170001. For the *R. helvetica* GEM, see MODEL2404170002.

## SUPPLEMENTAL MATERIAL

File S1 msystems.00507-25-s0001.xlsx Information about the strains; coverage; BUSCO, ANI, and TERA analysis; genome accessions; eggNOG annotation; RickA; and co-linearity analysis.

File S2 msystems.00507-25-s0002.pdf Genome-scale modeling details.

File S3 msystems.00507-25-s0003.pdf fastANI visualization.

File S4 msystems.00507-25-s0004.pdf Phylogenetic tree of the mitochondrial *Ixodes ricinus*.

File S5 msystems.00507-25-s0005.pdf *Rickettsia helvetica* localization in adult female ticks.

ASM does not own the copyrights to Supplemental Material that may be linked to, or accessed through, an article. The authors have granted ASM a non-exclusive, world-wide license to publish the Supplemental Material files. Please contact the corresponding author directly for reuse.
