# Supplementary material for: Deep sequencing of 16 Ixodes ricinus ticks unveils insights into their interactions with endosymbionts
Source: mSystems. 2025 Jun 16;10(7):e00507-25. doi: 10.1128/msystems.00507-25 (PMC12282096; doi:10.1128/msystems.00507-25)
Supplement: File S2 — Genome-scale modeling details. [file msystems.00507-25-s0002.pdf]

Supplementary File 2: **Genome-scale modeling details**

We performed a permutation test to determine the probability of observing the degree of overlap between the essential reactions of the symbionts by chance. We identified essential reactions for each symbiont using single reaction deletion analysis in COBRApy, considering reactions whose deletion resulted in a growth rate below 1e-6 as essential. We compiled the lists of essential reactions for Symbiont A (Rickettsia helvetica) and Symbiont B (Midochloria mitochondrii) and generated 10,000 randomized pairs of essential reaction sets by randomly sampling reactions from the combined reaction pool while preserving the original sizes of each set. For each permutation, we calculated the number of overlapping essential reactions and determined the p-value as the proportion of permutations where the overlap was greater than or equal to the observed overlap. Our analysis revealed that the number of overlapping essential reactions between Symbiont A and Symbiont B was 10. The average overlap in the permuted datasets was 6.68, with a standard deviation of 2.40. The permutation test yielded a p-value of 0.1221, indicating that the observed overlap is not statistically significant ($p > 0.05$). This suggests that the observed overlap of essential reactions is within the range expected by random chance, indicating neither significant complementarity (less overlap) nor redundancy (more overlap) in essential reactions between the symbionts at the reaction level. The lack of significant overlap implies that while the symbionts may share some essential reactions, their essential metabolic functions are largely independent. This may reflect an evolutionary balance between maintaining essential metabolic capabilities and relying on the host or each other for certain functions. These findings complement our previous analyses and suggest that metabolic complementarity may be more nuanced or occur at different levels (e.g., gene level, pathway level) not captured solely by essential reaction overlap.

Table S2.1. Statistical Results of the Permutation Test for Overlap in Essential Reactions

Statistic	Value
Observed overlap in essential reactions	10
Mean permuted overlap	6.68
Standard deviation of permuted overlaps	2.40
P-value from permutation test	0.1221

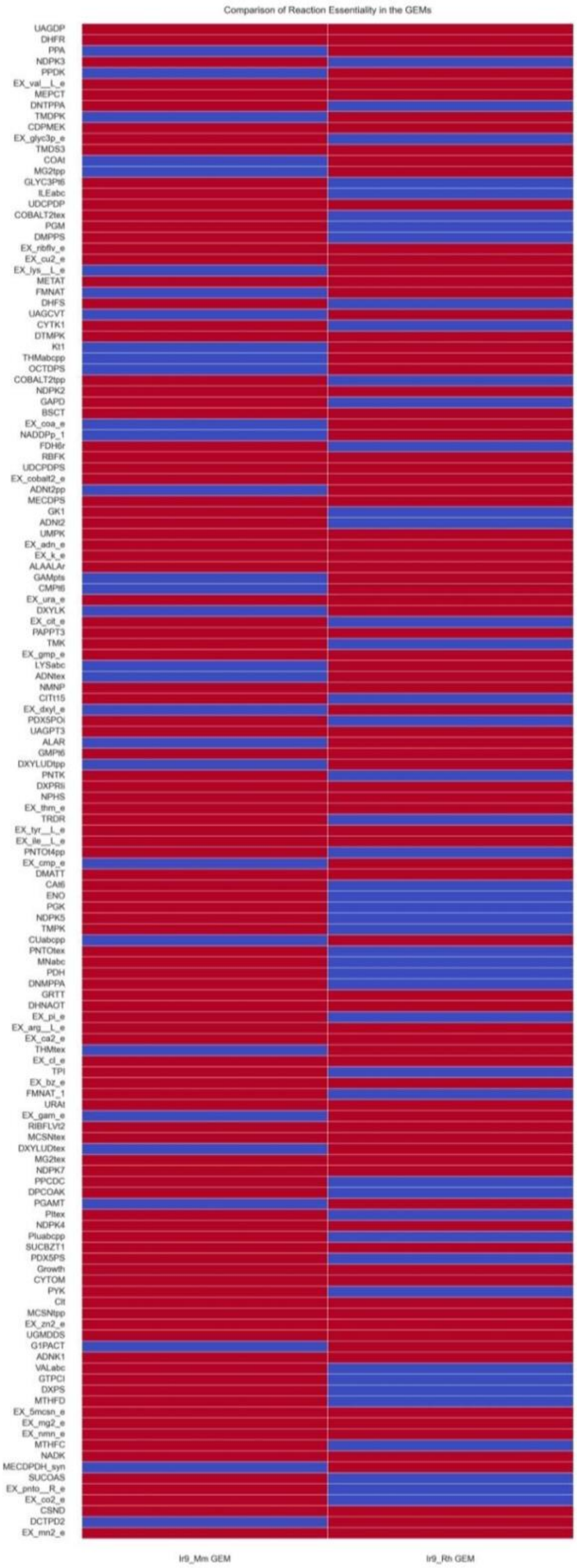

Figure S2.1: The genome-scale metabolic reconstructions and analysis for *M. mitochondrii* and *R. helvetica*, highlighting the essential reactions in red and the non-essential reactions in blue.

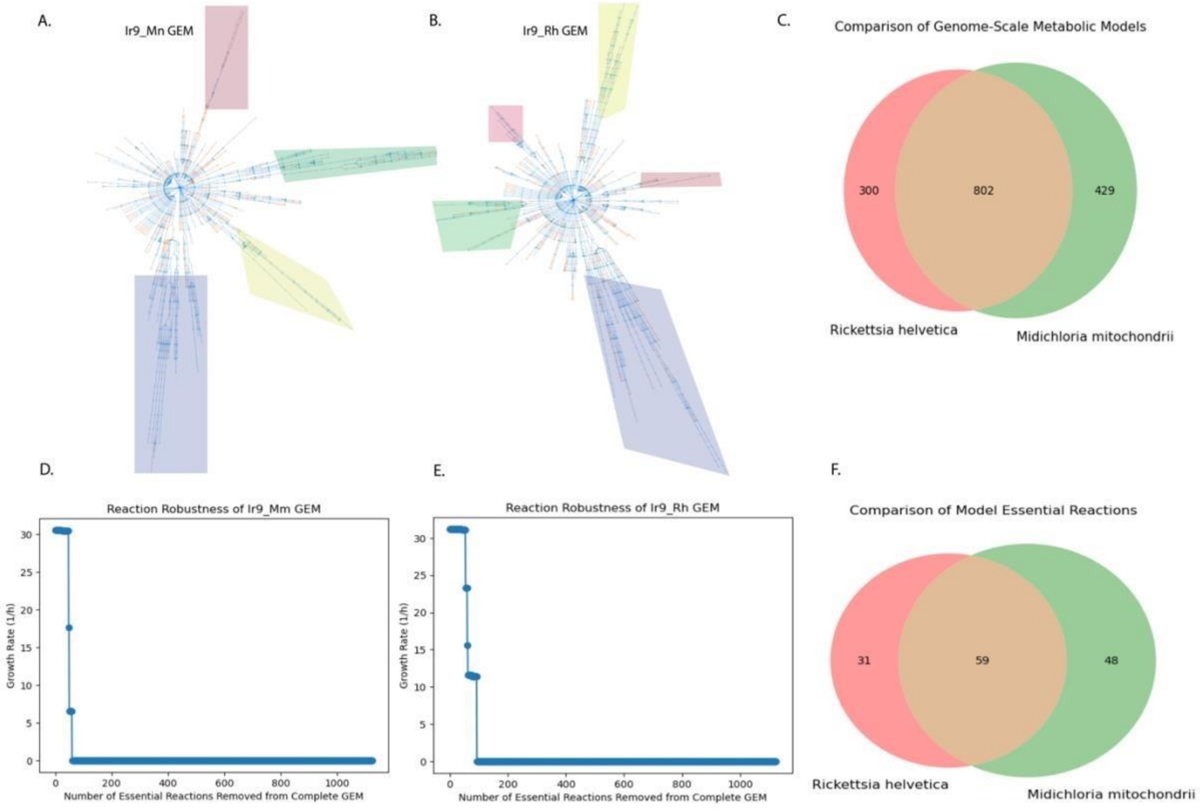

Figure S2.2: Genome-scale metabolic constructions and analysis of *M. mitochondrii* and *R. helvetica*. This figure presents genome-scale metabolic flux network maps of *M. mitochondrii* (Ir_d9_Mm GEM) in panel A, and *R. helvetica* (Ir9_Rh GEM) in panel B. The panels are color-coded: yellow for fatty acid metabolism, blue for phospholipid metabolism, green for central carbon metabolism, and red for amino acid & nitrogen metabolisms, visualized with Fluxer version 2.1 (Hari-2020-347). It also includes a Venn diagram comparing the reactions present within the Ir_d9_Rh GEM and Ir_d9_Mm GEM in panel C, reaction robustness analysis of the metabolic networks for *M. mitochondrii* (Ir_d9_Mm GEM) in panel D, and *R. helvetica* (Ir_d9_Rh GEM) in panel E, along with another Venn diagram comparing the reactions present within the Ir_d9_Rh GEM and Ir_d9_Mm GEM in panel F.

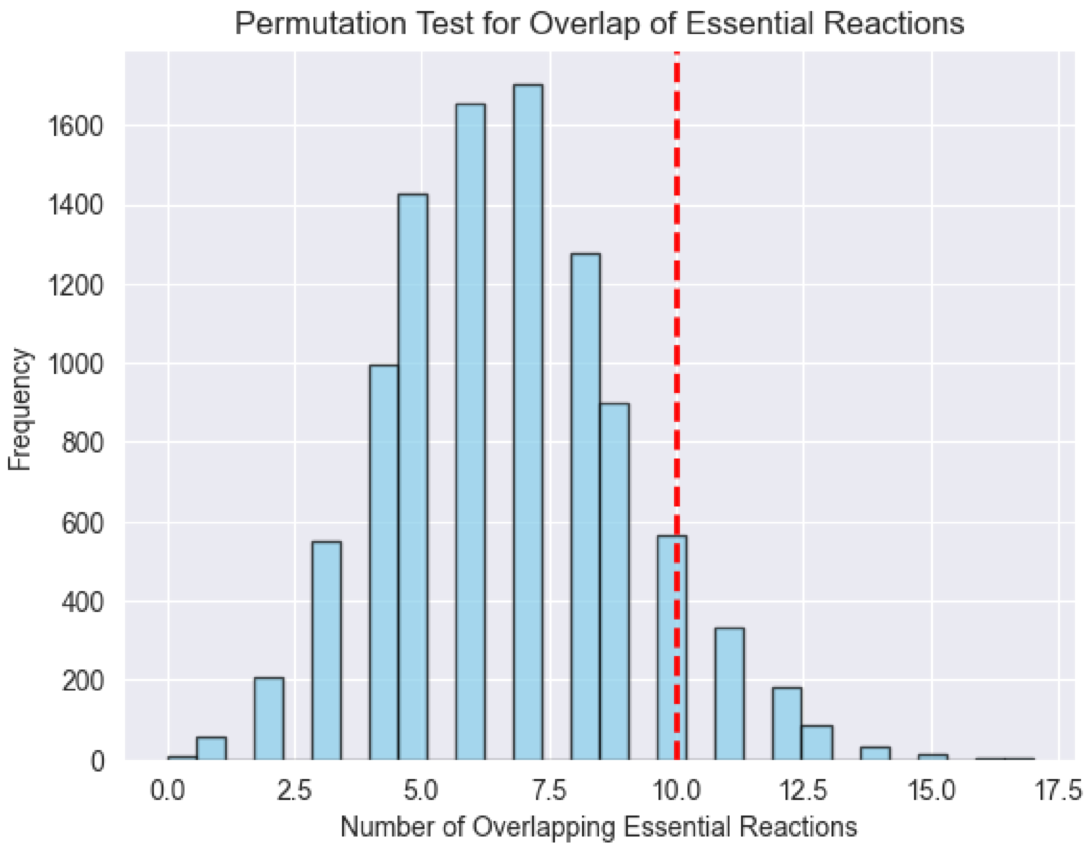

Figure S2.3: Histogram illustrating the distribution of overlaps from the permuted datasets, with the observed overlap indicated.
