# Supplementary material for: Deep sequencing of 16 Ixodes ricinus ticks unveils insights into their interactions with endosymbionts
Source: mSystems. 2025 Jun 16;10(7):e00507-25. doi: 10.1128/msystems.00507-25 (PMC12282096; doi:10.1128/msystems.00507-25)
Supplement: File S4 — Phylogenetic tree of the mitochondrial Ixodes ricinus. [file msystems.00507-25-s0004.pdf]

Reconstruction and phylogenetic analysis of the *I. ricinus* mitochondrial genomes

MT genome size ranged from 14562 to 14575 bp. For all 16, a circular genome was obtained coding for 13 protein encoding genes, 22 transfer RNA genes (tRNAs) and 2 ribosomal RNA genes (rRNAs) (Supplementary File 1). The AT content of the MT genomes ranged from 79.2% to 79.3%, a characteristic feature shared with other hard tick species [76]. A multiple alignment of the 16 MT sequences indicated 214 variable positions of which 147 were informative parsimony sites (excluding indels) and a mean nucleotide diversity of 0.00460. These variable sites defined a total of 15 haplotypes (mean haplotype diversity, Hd = 0.9917, with samples Ir f1 and Ir f3 carrying the same haplotype. The phylogenetic analysis of the mitogenomic data suggests that a locality-dependent structure based on geographic location (dune and forest) is absent (supplementary figure 2).

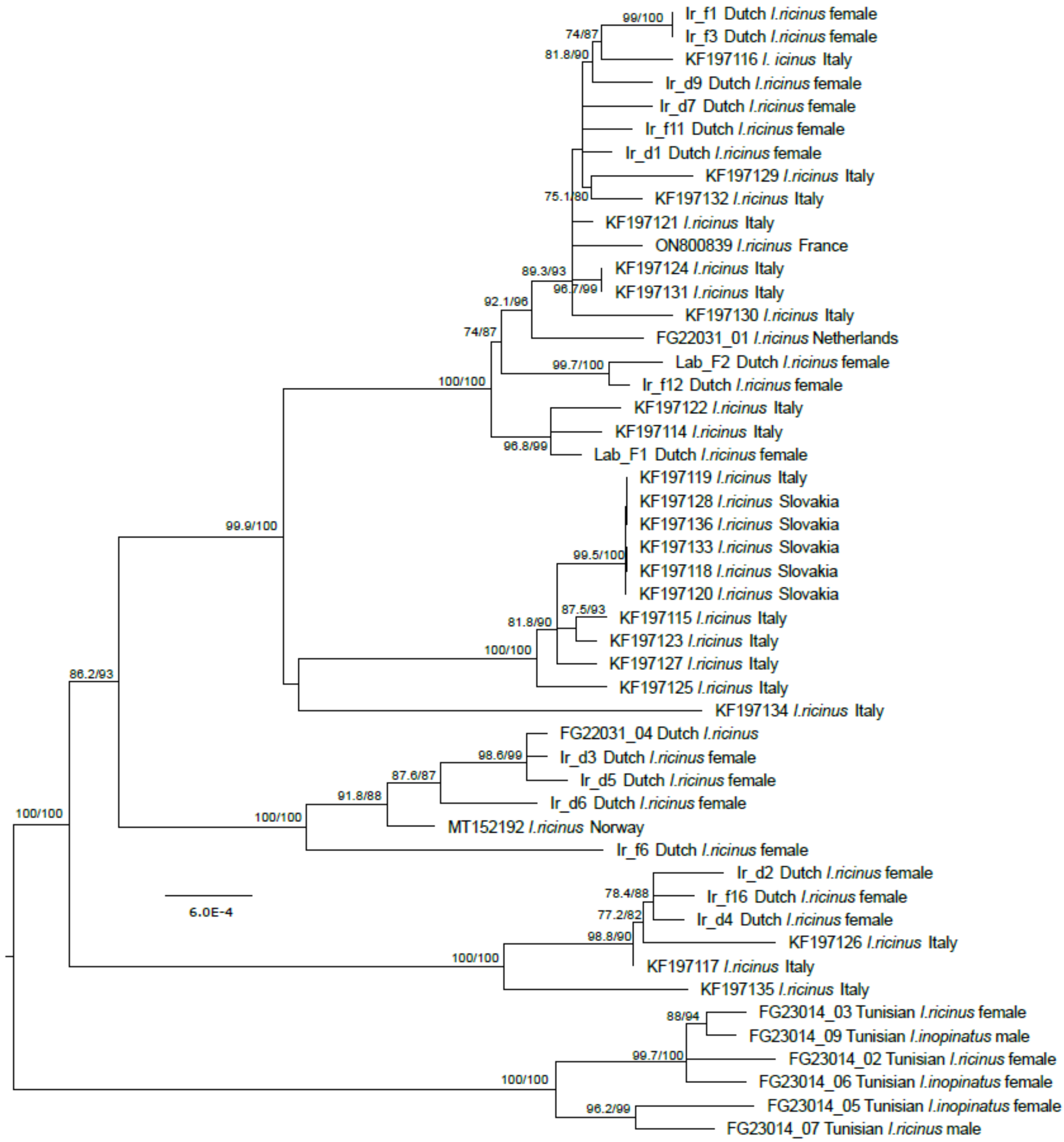

Fig 4.1. The phylogenetic tree of Ixodes ricinus based on the complete mt genome sequences of 16 *Ixodes ricinus* ticks collected in two localities (d-dune and f-forest) for the need of this study, and additional 33 mt genomes of *Ixodes ricinus / inopinatus* group deposited in the GenBank. The alignment was calculated using the CLUSTAL Omega implemented in the Geneious 11.1.4 software (Kearse et al., 2012).The tree was constructed using ML analysis implemented in the IQ-TREE software (Minh et al., 2020), and rooted based on a midpoint. The bootstrap values (SH-aLRT/UFB) above the 70/70 threshold are displayed. The scale bar indicates the number of nucleotide substitutions per site. Best-fit model according to BIC: K3Pu+F+I, was chosen based on the Bayesian

information criterion (BIC) computed by implemented ModelFinder (Kalyaanamoorthy et al., 2017). Branch supports were assessed by the ultrafast bootstrap (UFBoot) approximation (Minh et al., 2013) and by the SH-like approximate likelihood ratio test (SH- aLRT) (Guindon et al., 2010). The two sequences of I. persulcatus used as an outgroup are not displayed. The tree was visualized and graphically edited using FigTree v1.4.1

Input data: 49 sequences with 14634 nucleotide sites
Number of constant sites: 14159 (= 96.7541% of all sites)
Number of invariant (constant or ambiguous constant) sites: 14159 (= 96.7541% of all sites)
Number of parsimony informative sites: 284
Number of distinct site patterns: 395

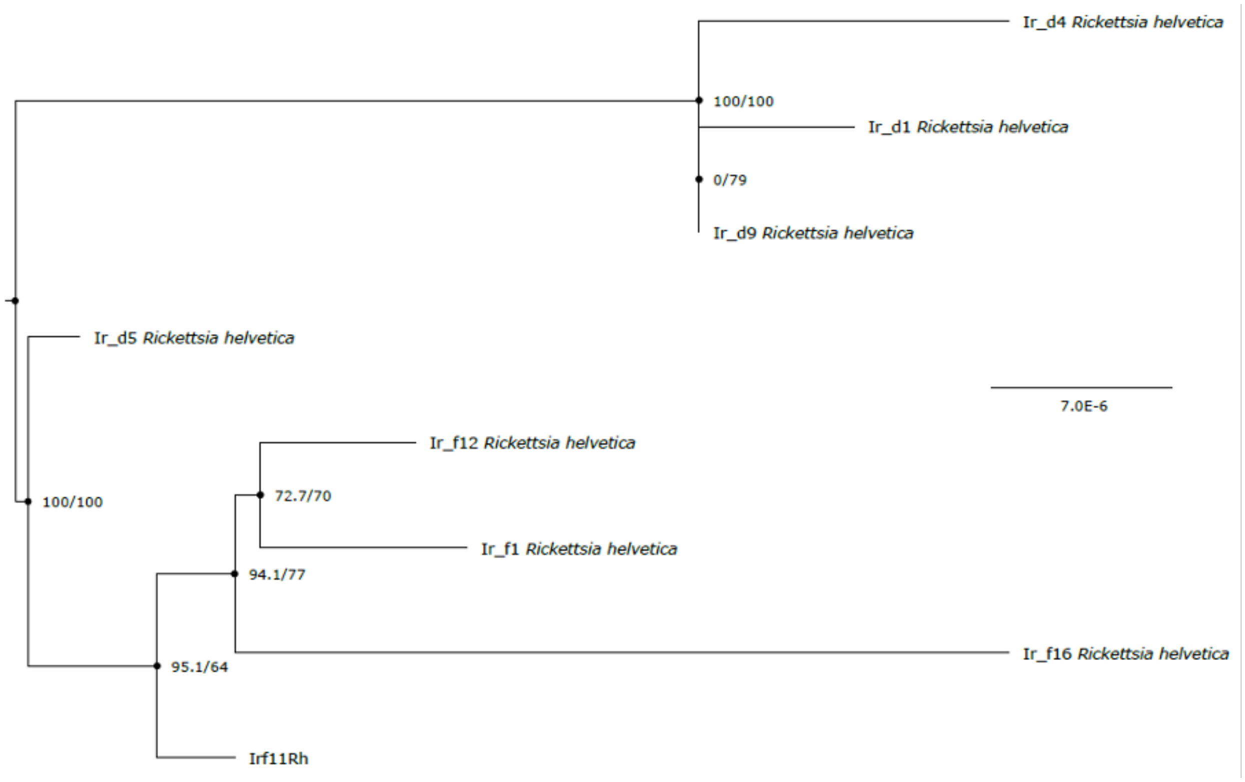

Fig 4.2. The phylogenetic tree of 8 R. helvetica partial genomes extracted from I. ricinus ticks collected in two localities (d-dune and f-forest) for the need of this study. For Rickettsia helvetica, we identified single-copy core genes (SCGs) using Anvi'o 7. Specifically, 1484 SCG clusters were identified for R. helvetica, with the length of 1011676 nt Gene alignments from SCGs were extracted and concatenated using the program "anvi-get-sequences-for-gene-clusters" with additional commands: --concatenate-gene-cluster and --report-DNA-sequences. The alignment was calculated using the CLUSTAL Omega implemented in the Geneious 11.1.4 software (Kearse et al., 2012).The tree was constructed using ML analysis implemented in the IQ-TREE software (Minh et al., 2020), and rooted based on a midpoint. The bootstrap values (SH-aLRT/UFB) above the 70/70 threshold are displayed. The scale bar indicates the number of nucleotide substitutions per site. Best-fit model according to BIC: HKY+F+I, was chosen based on the Bayesian information criterion (BIC) computed by implemented ModelFinder (Kalyaanamoorthy et al., 2017). Branch supports were assessed by the ultrafast bootstrap (UFBoot) approximation (Minh et al., 2013) and by the SH-like approximate likelihood ratio test (SH-aLRT) (Guindon et al., 2010). The two sequences of the tree was visualized and graphically edited using FigTree v1.4.1.

Input data: 8 sequences with 1011676 nucleotide sites Rickettsia helvetica
Number of constant sites: 1.01158e+06 (= 99.9908% of all sites)
Number of invariant (constant or ambiguous constant) sites: 1.01158e+06 (= 99.9908% of all sites)
Number of parsimony informative sites: 35
Number of distinct site patterns: 96

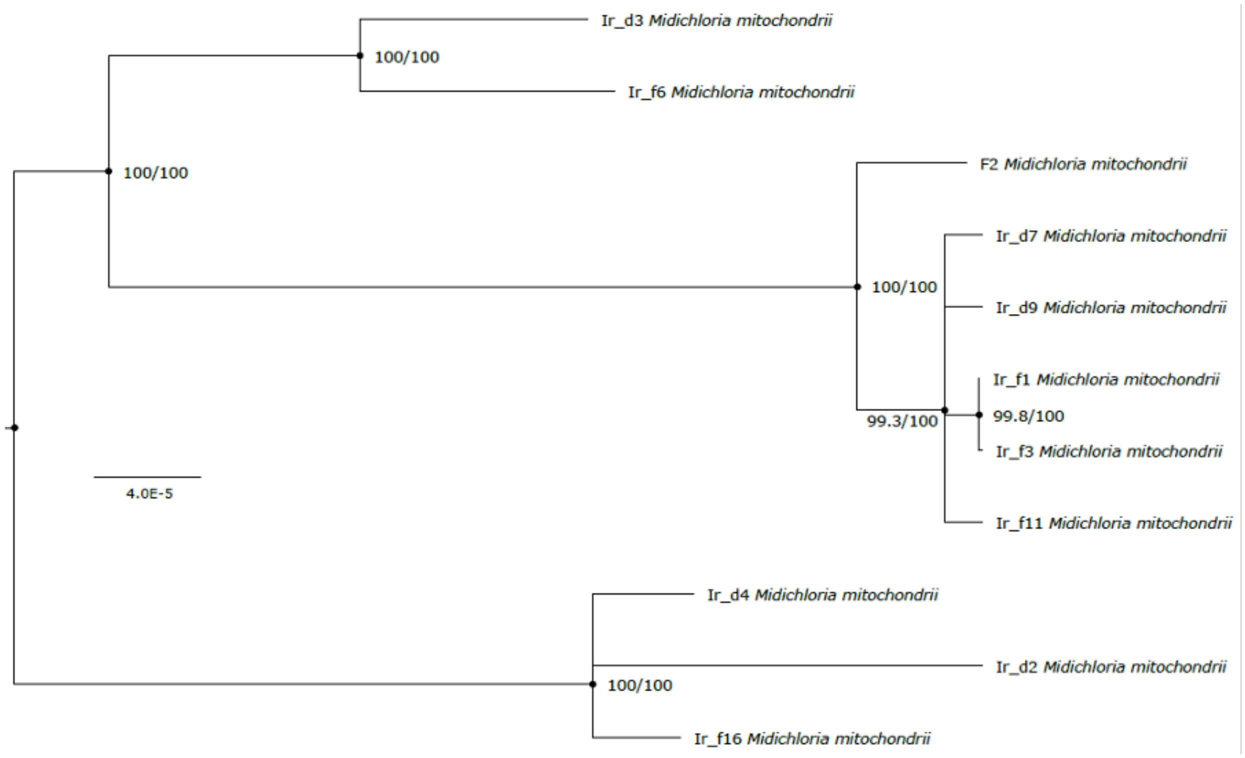

Fig. 4.3 The phylogenetic tree of 8 *M. mitochondrii* partial genomes extracted from I. ricinus ticks collected in two localities (d-dune and f-forest) for the need of this study. For Specifically, 1127 SCG clusters were identified for *M. mitochondrii*, with the length of 751501 nt, Gene alignments from SCGs were extracted and concatenated using the program "anvi-get-sequences-for-gene-clusters" with additional commands: --concatenate-gene-cluster and --report-DNA-sequences. The alignment was calculated using the CLUSTAL Omega implemented in the Geneious 11.1.4 software (Kearse et al., 2012). The tree was constructed using ML analysis implemented in the IQ-TREE software (Minh et al., 2020) and rooted based on a midpoint. The bootstrap values (SH-aLRT/UFB) above the 70/70 threshold are displayed. The scale bar indicates the number of nucleotide substitutions per site. Best-fit model according to BIC: HKY+F+I, was chosen based on the Bayesian information criterion (BIC) computed by implemented ModelFinder (Kalyaanamoorthy et al., 2017). Branch supports were assessed by the ultrafast bootstrap (UFBoot) approximation (Minh et al., 2013) and by the SH-like approximate likelihood ratio test (SH- aLRT) (Guindon et al., 2010). The two sequences of the tree were visualized and graphically edited using FigTree v1.4.1

Input data: 11 sequences with 751505 nucleotide sites Midichloria mitochondrii
Number of constant sites: 750660 (= 99.8876% of all sites)
Number of invariant (constant or ambiguous constant) sites: 750660 (= 99.8876% of all sites)
Number of parsimony informative sites: 392
Number of distinct site patterns: 376
Best-fit model according to BIC: K3Pu+F+I
