# Supplementary material for: Deep sequencing of 16 Ixodes ricinus ticks unveils insights into their interactions with endosymbionts
Source: mSystems. 2025 Jun 16;10(7):e00507-25. doi: 10.1128/msystems.00507-25 (PMC12282096; doi:10.1128/msystems.00507-25)
Supplement: File S5 — Rickettsia helvetica localization in adult female ticks. [file msystems.00507-25-s0005.pdf]

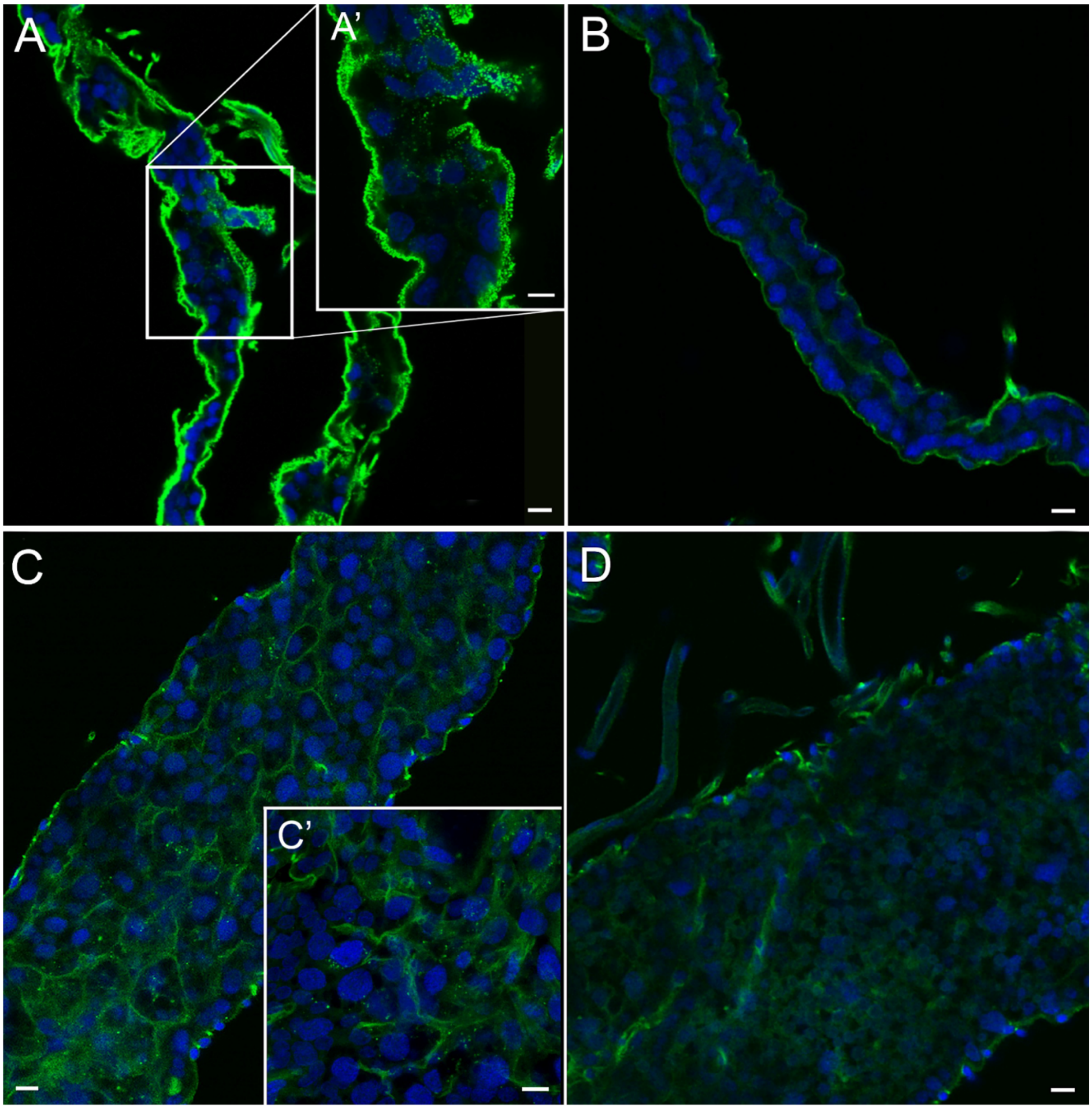

Supplementary Figure 5.1: Blue: DAPI, Green dots: *Rickettsia* specific binding. The scale bars represent 10 µM. A) *R. helvetica* positive malpighian tubule X40 A') *R. helvetica* positive malpighian tubule X100, B) Control malpighian tubule , C) *R. helvetica* positive gut X40 C') *R. helvetica* positive gut X100, D) Control gut.

*Green tissue non-specific autofluoresence can be seen in the periphery of treatments and controls as is often seen in immunostaining.

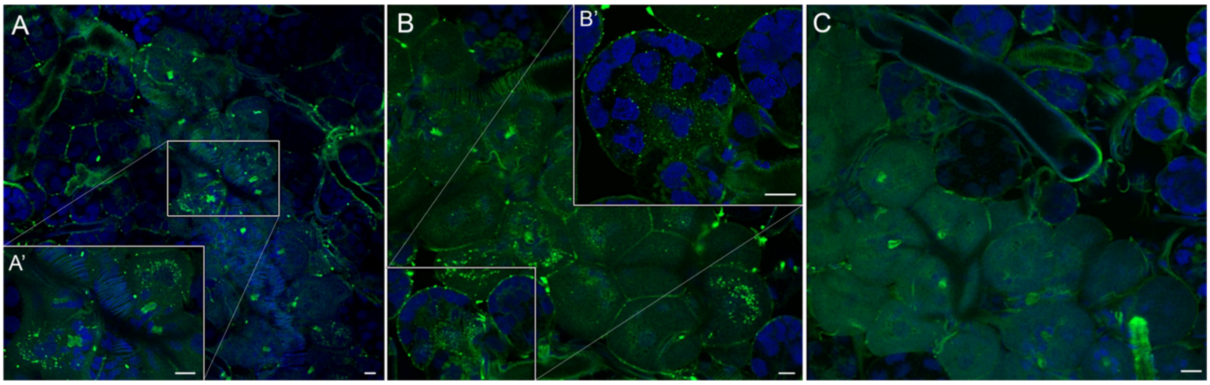

Supplementary Figure 5.2: Blue: DAPI, Green dots: *Rickettsia* specific binding. The scale bars represent 10 µM. A) *R. helvetica* positive salivary glands type I X40 A') *R. helvetica* positive salivary glands type I X100, B) *R. helvetica* positive salivary glands type II X40, B') *R. helvetica positive* salivary gland type II X100, C) Control salivary glands.

*Green tissue non-specific autofluoresence can be seen in the periphery of treatments and controls as is often seen in immunostaining.
