# Supplementary material for: Deep sequencing of 16 Ixodes ricinus ticks unveils insights into their interactions with endosymbionts
Source: mSystems. 2025 Jun 16;10(7):e00507-25. doi: 10.1128/msystems.00507-25 (PMC12282096; doi:10.1128/msystems.00507-25)
Supplement: File S3 — fastANI visualization. [file msystems.00507-25-s0003.pdf]

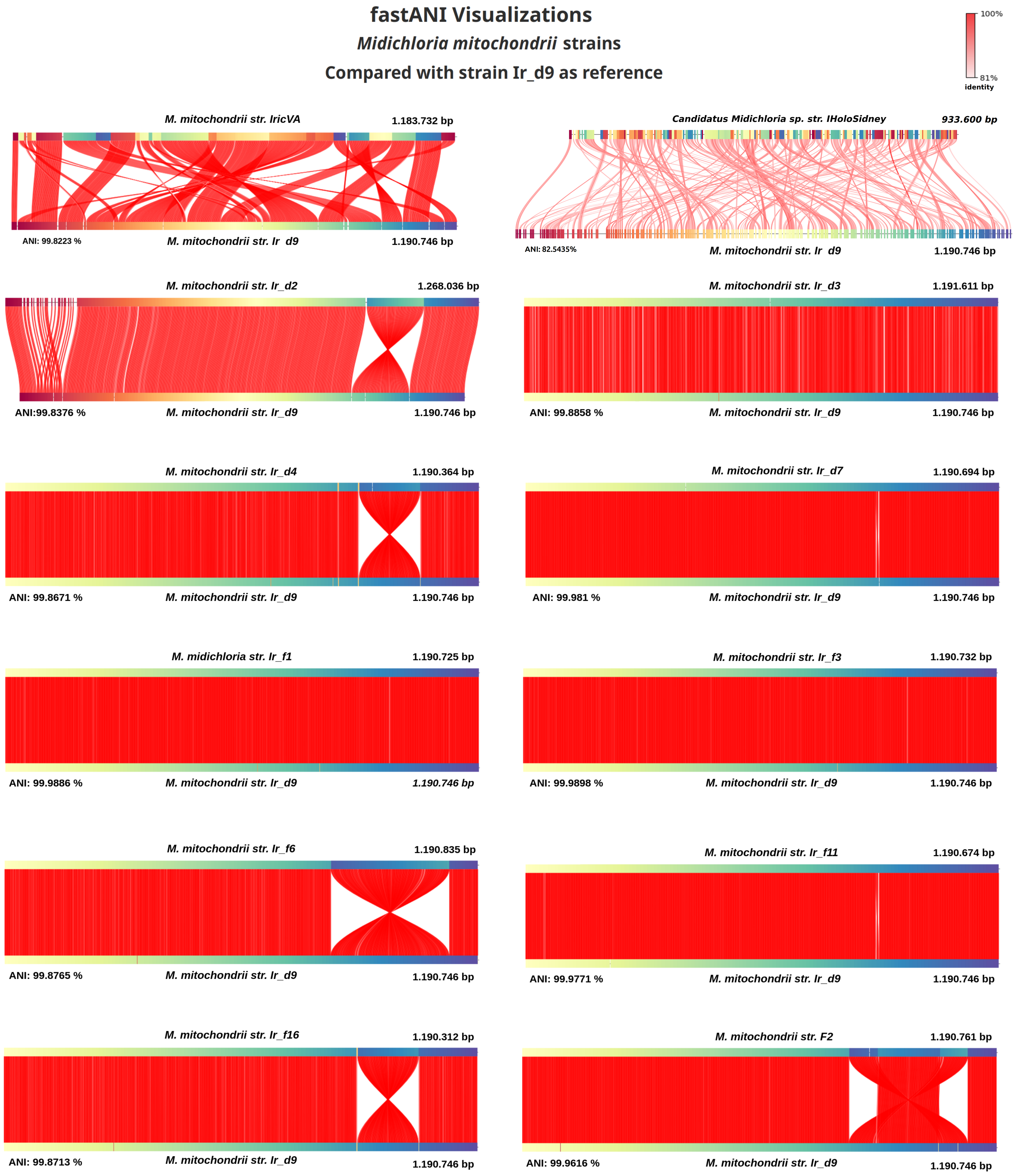

Figure 3.1: Visualized FastANI comparison using strain M. mitochondrii str. Ir_d9 as reference against all other strains obtained in this study. Additionally, a comparison was performed with M. mitochondrii str. IricVA and Ca.Midichloria sp. str IholoSydney. Genomic rearrangements were observed in five strains (Ir_d2, Ir_d4, Ir_f6, Ir_f16, and F2), which align with the patterns observed in the mitochondrial phylogenetic tree.
